# Structural changes during water-mediated amorphization of semiconducting two-dimensional thio­stannates

**DOI:** 10.1107/S2052252519006791

**Published:** 2019-07-05

**Authors:** Mathias S. Hvid, Henrik S. Jeppesen, Matteo Miola, Paolo Lamagni, Ren Su, Kirsten M. Ø. Jensen, Nina Lock

**Affiliations:** a Interdisciplinary Nanoscience Center (iNANO), Aarhus University, Gustav Wieds Vej 14, Aarhus C DK-8000, Denmark; b Sino-Danish Center for Education and Research (SDC), Interdisciplinary Nanoscience Center (iNANO), Aarhus University, Gustav Wieds Vej 14, Aarhus C DK-8000, Denmark; c Carbon Dioxide Activations Center (CADIAC), Interdisciplinary Nanoscience Center (iNANO), Aarhus University, Gustav Wieds Vej 14, Aarhus C DK-8000, Denmark; d SynCat@Beijing, Synfuels China Technology Co. Ltd., Leyuan South Street II, No.1, Yanqi Economic Development Zone C#, Huairou District, Beijing 101407, People’s Republic of China; e Department of Chemistry, University of Copenhagen, Universitetsparken 5, København Ø 2100, Denmark; f Carbon Dioxide Activations Center (CADIAC), Interdisciplinary Nanoscience Center (iNANO) and Department of Chemistry, Aarhus University, Gustav Wieds Vej 14, Aarhus C DK-8000, Denmark

**Keywords:** two-dimensional thiostannates, total scattering, pair distribution function analysis, semiconductors, amorphization

## Abstract

Owing to their combined open-framework structures and semiconducting properties, two-dimensional thio­stannates show great potential for catalytic and sensing applications. One such class of crystalline materials consists of porous polymeric [Sn_3_S_7_^2−^]*_n_* sheets with molecular cations embedded in-between. The compounds are denoted *R*-SnS-1, where *R* is the cation. Dependent on the cation, some *R*-SnS-1 thio­stannates transition into amorphous phases upon dispersion in water. Knowledge about the fundamental chemical properties of the thio­stannates, including their water stability and the nature of the amorphous products, has not yet been established. This paper presents a time-resolved study of the transition from the crystalline to the amorphous phase of two violet-light absorbing thio­stannates, *i.e.* AEPz-SnS-1 [AEPz = 1-(2-amino­ethyl)­piperazine] and trenH-SnS-1 [tren = tris­(2-amino­ethyl)­amine]. X-ray total scattering data and pair distribution function analysis reveal no change in the local intralayer coordination during the amorphization. However, a rapid decrease in the crystalline domain sizes upon suspension in water is demonstrated. Although scanning electron microscopy shows no significant decrease of the micrometre-sized particles, transmission electron microscopy reveals the formation of small particles (∼200–400 nm) in addition to the larger particles. The amorphization is associated with disorder of the thio­stannate nanosheet stacking. For example, an average decrease in the interlayer distance (from 19.0 to 15.6 Å) is connected to the substantial loss of the organic components as shown by elemental analysis and X-ray photoelectron spectroscopy. Despite the structural changes, the light absorption properties of the amorphisized *R*-SnS-1 compounds remain intact, which is encouraging for future water-based applications of such materials.

## Abstract 2

The amorphization of semiconducting two-dimensional thio­stannates was studied using X-ray total scattering and pair distribution function analysis. The local structure and light absorption properties are retained, while the amorphization is associated with disorder of the thio­stannate nanosheet stacking.

## 1. Introduction   

Porous thio­metallates are an interesting group of compounds due to their combined open frameworks and semiconducting properties (Seidlhofer *et al.*, 2010 ▸; Wu *et al.*, 2015*a* ▸; Tang *et al.*, 2018 ▸). They share some structural similarities with zeolites, *i.e.* a family of microporous aluminosilicates, which have provided enormous utility in the chemical industry owing to their ion exchange, gas separation and catalytic capabilities (Holm *et al.*, 2011 ▸; Martínez & Corma, 2011 ▸; Kosinov *et al.*, 2016 ▸). Combining these chemical properties with the semiconducting nature of thio­metallates is highly intriguing, and may expand the application of sulfide-based porous materials to new areas such as photocatalysis (Chen *et al.*, 2018 ▸; Lin *et al.*, 2015 ▸; Shim *et al.*, 2013 ▸). In this regard, the utilization of thio­stannates is promising, and a number of such compounds have been reported, see *e.g.* Jiang & Ozin (1998 ▸), Jiang *et al.* (1998*a* ▸) and Seidlhofer *et al.* (2010 ▸). Tetrahedral [SnS_4_]^4−^ units form the fundamental building blocks in thio­stannates, and depending on the connectivity of these subunits, discrete complexes or extended networks of one-dimensional chains (Baiyin *et al.*, 2004 ▸) or two-dimensional layers (Ko *et al.*, 1994 ▸) are formed.

Naturally occurring binary tin sulfides, *i.e.* SnS and SnS_2_, are both layered materials consisting of densely packed trigonal pyramidal SnS_3_ and octahedral SnS_6_ clusters, respectively. Photocatalytic properties under visible light irradiation have been reported for both compounds (Jing *et al.*, 2018 ▸; Wu *et al.*, 2015*b* ▸; Tang *et al.*, 2011 ▸). By distorting the Sn:S ratio and introducing organic components into the synthesis, two-dimensional thio­stannates with porous layers and larger accessible surface areas can be formed. For instance, a material consisting of [Sn_3_S_7_^2−^]*_n_* layers with 24-atom hexagonal pores results from solvothermal synthesis using tetra­methyl­ammonium (TMA) as the structure-directing agent (Parise *et al.*, 1994 ▸). Charge stabilizing cationic TMA is embedded in-between the anionic polymeric thio­stannate layers, and electrostatic interactions hold together the structure. For comparison, the bulky structure-directing agent tetra­propyl­ammonium (TPA) produces larger pores (32-atom) in an [Sn_4_S_9_^2−^]*_n_* layered compound with TPA as the counter ion (Ko *et al.*, 1995 ▸).

Bedard and coworkers studied several aspects of the synthesis, structure and properties of layered thio­stannates (Jiang *et al.*, 1998*b* ▸,*a* ▸,*c* ▸ Bowes *et al.*, 1998 ▸), and they introduced the notation *R*-SnS-*n*, where *R* denotes the cation, and *n* is used to distinguish between different structure types. *R*-SnS-1 refers to the [Sn_3_S_7_^2−^]*_n_* structure type in which the porous thio­stannate layers attain a hexagonal honeycomb-like structure. Sn_3_S_4_ broken-cube clusters form the basic structural building block in the [Sn_3_S_7_^2−^]*_n_* layers, where each cluster connects to its neighbors by three double sulfur bridges. The *R*-SnS-1 compounds have semiconducting properties and are violet-light absorbers with band gaps of approximately 3 eV (Filsø *et al.*, 2017 ▸; Qi *et al.*, 2015 ▸). The combination of an open-framework structure and semiconducting properties make these compounds interesting for catalytic and sensing applications (Jiang *et al.*, 1998*b* ▸).

The molecular cations residing between the thio­stannate layers are typically organic quaternary ammonium or protonated amine species. Since it is possible to exchange these cations in solution, *R*-SnS-1 materials have recently attracted attention as potential ion exchangers for heavy metals and molecular organic cations (Feng *et al.*, 2016 ▸; Qi *et al.*, 2017 ▸, 2015 ▸; Hvid *et al.*, 2017 ▸). The cations are often crystallographically ordered, *i.e.* located at specific sites within the thio­stannate framework, as is the cases of, for example, TMA-SnS-1 (Parise *et al.*, 1994 ▸), DABCOH-SnS-1 (DABCOH = protonated 1,4-di­aza­bicyclo­[2.2.2]octane; Jiang *et al.*, 1998*c* ▸) and 1AEP-SnS-1 [1AEP = 1-(2-amino­ethyl)­piperidine; Filsø *et al.*, 2017 ▸]. However, a few examples exist where the cations are crystallographically disordered, notably trenH-SnS-1 [trenH = protonated tris­(2-amino­ethyl)­amine; Pienack *et al.*, 2012 ▸].

We have previously reported on the structural instability of trenH-SnS-1 upon dispersion in water (Hvid *et al.*, 2017 ▸). The crystalline structure of the thio­stannate transforms to an amorphous phase in aqueous solution within 1 h. Similar structural instability has been observed for the compound AEPz-SnS-1 [AEPz = 1-(2-amino­ethyl)­piperazine; Walther *et al.*, 2019 ▸], which is structurally closely related to trenH-SnS-1. However, the chemical nature of the amorphous products has not previously been studied, and the phase transition is poorly understood. Recently, there has been increasing interest in fundamental studies and applications of amorphous functional materials, *e.g.* battery materials (Zhang *et al.*, 2016 ▸; Jiangfeng *et al.*, 2013 ▸), optical materials (Rosemann *et al.*, 2016 ▸) and catalysts (Yuyang *et al.*, 2015 ▸; Smith *et al.*, 2013 ▸; Benck *et al.*, 2012 ▸). The properties of amorphous compounds are often distinctly different from those of their crystalline counterparts; while the surface area and accessibility of active sites may be increased by amorphization, physical properties such as electric and thermal conductivities are also likely to be affected (Siegrist *et al.*, 2011 ▸; Hosseini *et al.*, 2014 ▸). Therefore, knowledge of the structure is the key to understanding structure–property relationships and to rationally design advanced amorphous materials.

**Figure 1:**
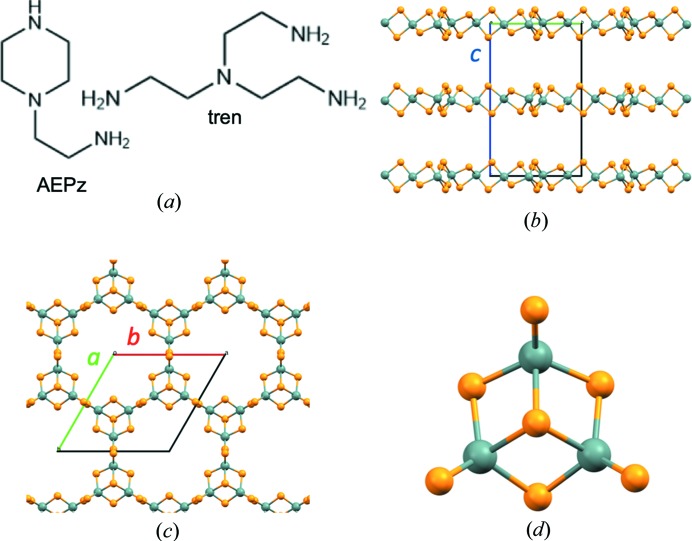
Structures of AEPz-SnS-1 and trenH-SnS-1: (*a*) Molecular structures of AEPz and tren (shown in their unprotonated forms). (*b*) Side-view showing the thio­stannate layers stacked along the crystallographic *c* axis. (*c*) Top-view of a porous thio­stannate layer. (*d*) Fundamental building block of the *R*-SnS-1 structures, *i.e.* the Sn_3_S_4_ broken-cube cluster, which is connected to three neighboring clusters through double sulfur bridges. Sn is shown in gray and sulfur is shown in orange. The structural drawings are based on the single-crystal structure of trenH-SnS-1 (Filsø *et al.*, 2017 ▸).

Here we report the study of the amorphization of trenH-SnS-1 and AEPz-SnS-1 (Fig. 1 ▸) upon dispersion in water. X-ray total scattering (TS) and pair distribution function (PDF) analysis have been used to study the atomic scale structure of samples subjected to water treatment for different durations, providing information about the structural changes over time. The powder X-ray diffraction (PXRD) patterns change substantially over time, whereas the real-space time-resolved PDFs show no change in the local tin sulfide coordination, pointing to preservation of the fundamental structural motifs (*i.e.* the Sn_3_S_4_ broken cube clusters) in the thio­stannate sheets. Structural details such as the average domain size and interlayer distance have been extracted from analysis of PXRD and PDF data. The results are compared with electron microscopy images of the samples and the chemical composition as shown by X-ray photoelectron spectroscopy (XPS) and elemental analysis (CHNS). The optical properties of the amorphous products were investigated using diffuse reflectance spectroscopy, revealing that the optical band gaps remain largely unchanged despite substantial structural alterations.

## 2. Experimental   

### 2.1. Chemicals   

Chemical precursors SnO_2_ (≥99.9%), sulfur (≥99.5%), tris­(2-amino­ethyl)­amine (C_6_H_18_N_4_, tren, 96%) and 1-(2-amino­ethyl)­piperazine (C_6_H_15_N_3_, AEPz, 99%) were obtained from Sigma–Aldrich and used without further purification.

### 2.2. Synthesis   

#### 2.2.1. Synthesis of trenH-SnS-1   

The synthesis of trenH-SnS-1 was carried out as described previously (Hvid *et al.*, 2017 ▸), but in this case, using an Sn:S ratio of 3:8 to minimize SnO_2_ precursor impurities. A mass of 800 mg SnO_2_ and 450 mg elemental sulfur were treated solvothermally in 4 ml tren at 190°C for 6 days in a Teflon-lined (22 ml) stainless steel autoclave. The product was isolated by filtration and washed with aceto­nitrile. A yield of ∼1.2 g [*i.e.* 77% based on Sn in Sn_3_S_7_(trenH)_2_] was obtained.

#### 2.2.2. Synthesis of AEPz-SnS-1   

AEPz-SnS-1 was recently reported (Walther *et al.*, 2019 ▸). Herein, we synthesized the compound using a slightly modified two-step solvothermal protocol to minimize SnO_2_ impurities. Step 1: a mixture of 400 mg SnO_2_ and 200 mg sulfur (Sn:S molar ratio of 3:7) was ground and placed in a 22 ml Teflon-lined stainless steel autoclave with 2 ml AEPz. The mixture was heated for 6 days at 190°C. The product, *i.e.* AEPz-SnS-1 with SnO_2_ leftovers, was isolated by suction filtration and washed with ethanol, yield: ∼0.74 g. Step 2: the powder from Step 1 was ground and sulfur was added (*i.e.* 170 mg sulfur per gram of product from Step 1). The powder mixture was treated solvothermally in 2 ml AEPz for another 6 days at 190°C before it was isolated and washed in ethanol to obtain a final yield of ∼0.5 g [*i.e.* 67% based on Sn in Sn_3_S_7_(AEPz)_2_].

#### 2.2.3. Water-mediated amorphization of trenH-SnS-1 and AEPz-SnS-1   

Water-treated samples were prepared by dispersing portions of 100–150 mg trenH-SnS-1 or AEPz-SnS-1 in 10 ml deionized H_2_O without stirring for a given duration *t*, such that each sample represented a specific point in time of the amorphization process. The samples were then quickly isolated by suction filtration and dried, thus halting the progression of amorphization. These filtered powders were analyzed as described below. Note that the smallest particles formed in this process may have been dispersed in the filtrate. Only minute amounts of these fine particles could be isolated, confirming their presence in low concentration.

### 2.3. Powder X-ray diffraction   

PXRD data were collected at room temperature on a Rigaku SmartLab diffractometer using Cu *K*α_1_ radiation. Data were measured in transmission geometry on ground powders packed in 0.4 mm glass capillaries. The diffracted intensities were collected using a D/TEX Ultra 256 multi-channel detector. Le Bail profile fitting was performed using the *FullProf* software (Rodríguez-Carvajal, 1993 ▸).

### 2.4. X-ray total scattering   

Total scattering data were collected (room temperature) at the P02.1 beamline at PETRA III of the Deutsches Elektronen-Synchrotron (DESY), Hamburg, Germany, with an X-ray energy of 60 keV (λ = 0.2072 Å). Powder samples were loaded in Kapton capillaries (inner diameter = 1.0 mm). Two-dimensional scattering images were acquired over 60 s, this process was repeated four times for each sample. The sample-to-detector distance of approximately 250 mm was calibrated using a CeO_2_ standard, resulting in a *Q*_max_ of 19.5 Å^−1^. Azimuthal integration and summation of the four detector frames for each sample were performed using *Dioptas* (v. 0.4; Prescher & Prakapenka, 2015 ▸). PDFs, *G*(*r*), were obtained by Fourier transformation of the integrated data in the *xPDFSuite* software (Yang *et al.*, 2014 ▸). The background scattering was corrected for by subtracting the scattered intensity of an empty Kapton capillary. Refinement of the PDF data was done in *PDFgui* (Farrow *et al.*, 2007 ▸) using a crystalline model of trenH-SnS-1 (Filsø *et al.*, 2017 ▸). The instrument dampening was determined from refinements of a CeO_2_ standard and included in the data modeling.

### 2.5. Scanning electron microscopy   

Scanning electron microscopy (SEM) images of trenH-SnS-1 and AEPz-SnS-1 before and after water treatment were acquired on an FEI-Nova Nano SEM 600 scanning electron microscope under high-vacuum conditions (3 × 10^−5^ mbar). Powders were immobilized on sticky carbon tape, and the samples were coated with a 10 nm gold layer using a LEICA EM SCD 500 vacuum film-deposition system equipped with a LEICA EM QSG100 Quartz Crystal Film Thickness Monitor.

### 2.6. Transmission electron microscopy   

Transmission electron microscopy (TEM) images were acquired on a Tecnai Spirit electron microscope equipped with a TWIN lens system operating at 120 kV, and using a Veleta CCD side-mounted camera. Filtered powder samples of trenH-SnS-1 (1 h in water) and AEPz-SnS-1 (24 h in water) were suspended in iso­propanol and mounted on carbon-coated copper grids.

### 2.7. X-ray photoelectron spectroscopy   

XPS analysis was performed using a Kratos Axis Ultra-DLD instrument equipped with an Al *K*α 150 W X-ray source. During the measurements the chamber pressure was kept below 5 × 10^−9^ mbar. Survey and high-resolution spectra were obtained with a pass energy of 120 and 20 eV, respectively. For each sample, spectra were measured at three different positions for statistical purposes. An electron flood gun charge neutraliser was used during the entire measurement. Deconvolution of the spectra was performed with the *CasaXPS* software. The energy Sn 3*d* 3/2 = 486.6 eV was used for the binding energy calibration based on the similar SnS_2_, which has been reported in the literature (Price *et al.*, 1999 ▸; Hyeongsu *et al.*, 2018 ▸; He *et al.*, 2013 ▸). The peaks were fitted with a GL(30) function except for Sn 3*d*, for which an asymmetric shape was used, *i.e.* an A(0.1,0.2,0) GL(60) function.

### 2.8. Elemental analysis   

Elemental analysis was performed on samples of pristine and water-treated (1 h) thio­stannates (AEPz-SnS-1 and trenH-SnS-1). An Elementar Vario MACRO cube instrument in CHNS mode was used for the analysis with sulfanilamide as a standard. Approximately 23 mg sulfanilamide or 32 mg thio­stannate was used per measurement. All thio­stannate samples have been measured three times.

### 2.9. Diffuse reflectance spectroscopy   

Light absorption properties of the thio­stannate compounds were examined by diffuse reflectance spectroscopy (DRS) on a Shimadzu UV-3600 spectrophotometer. Powder samples were distributed on a BaSO_4_ reference powder, and spectra were recorded between 200 and 1200 nm with a step size of 1 nm.

### 2.10. Solid-state nuclear magnetic resonance spectroscopy   

The solid-state ^13^C{^1^H} CP/MAS NMR spectrum of pristine AEPz-SnS-1 was obtained on a Bruker Avance II 400 MHz (9.4 T) spectrometer using a home-built 7 mm CP/MAS NMR probe, a spinning speed of ν_R_ = 4.0 kHz, an 8 s relaxation delay, a CP contact time of 0.5 ms and 8192 scans. ^13^C chemical shifts are referenced to tetra­methyl­silane (TMS).

## 3. Results and discussion   

The thio­stannate layers of trenH-SnS-1 and AEPz-SnS-1 are isostructural and crystallize in the hexagonal space group *P*6_3_/*mmc*. The two compounds consist of two-dimensional [Sn_3_S_7_^2−^]*_n_* thio­stannate sheets stacked in an ABAB sequence along the crystallographic *c* axis [Figs. 1 ▸(*b*) and 1(*c*)]. The layers consist of Sn_3_S_4_ broken cube clusters, which are connected *via* double sulfur bridges [Fig. 1 ▸(*d*)], and crystallographically disordered cations [Fig. 1 ▸(*a*)] are embedded between the layers. The compound trenH-SnS-1 has previously been reported to have the composition (trenH)_2_Sn_3_S_7_ (Filsø *et al.*, 2017 ▸; Hvid *et al.*, 2017 ▸), whereas the stoichiometry of AEPz-SnS-1 has been determined through elemental analysis to consist of two AEPz molecules per [Sn_3_S_7_^2−^]_*n*_ moiety (Table S10). In light of this, we propose that each AEPz molecule has been monoprotonated in order to charge compensate for the anionic layers. Despite the different nature of the structure-directing agents used in the synthesis of trenH-SnS-1 and AEPz-SnS-1, PXRD data of the two pristine compounds confirm the thio­stannate layer structures of trenH-SnS-1 and AEPz-SnS-1 to be identical. The double interplanar spacing, *i.e.* the *c* axis, is strikingly similar in the two compounds, as it is 19.2195 (5) and 19.0860 (8) Å for AEPz-SnS-1 and trenH-SnS-1, respectively, according to Le Bails refinements of the pristine compound PXRD data (Fig. S1) (Filsø *et al.*, 2017 ▸; Walther *et al.*, 2019 ▸). Solid-state NMR has furthermore confirmed the incorporation of AEPz into the structure (Fig. S11).

### 3.1. Loss of crystalline order   

In order to track the water-mediated amorphization of the compounds, PXRD and X-ray total scattering data were collected for series of samples, which had been dispersed in water for different durations.

**Figure 2:**
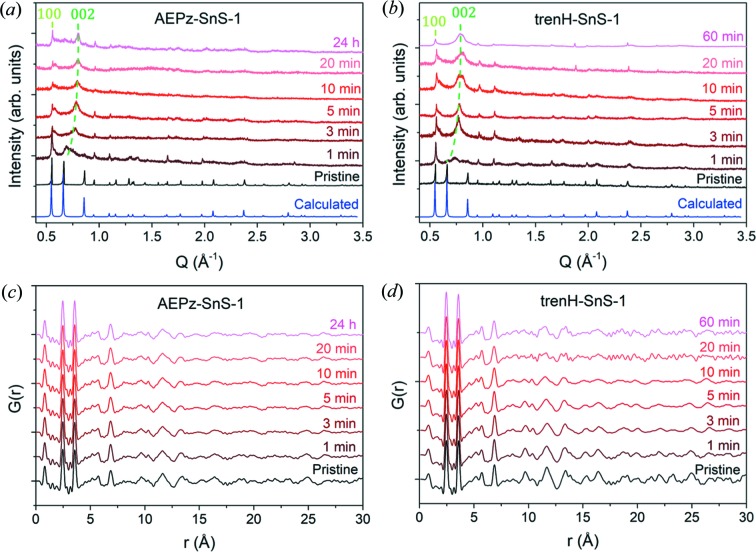
PXRD patterns of pristine and water-treated (*a*) AEPz-SnS-1 and (*b*) trenH-SnS-1. The positions of the (100) and (002) peaks of the pristine samples are shown. The peak at 0.86 Å^−1^ corresponds to (102). The calculated patterns are based on the crystal structure of trenH-SnS-1 (Filsø *et al.*, 2017 ▸). Peaks from a minor SnO_2_ impurity are observed at 1.88 and 2.38 Å^−1^ in the 20 and 60 min trenH-SnS-1 samples. The same batch of trenH-SnS-1 was used for preparation of all samples presented in Figs. 2 ▸(*b*) and 2 ▸(*d*), and the SnO_2_ peaks are barely observable except for samples with low thio­stannate crystallinity, *i.e.* 20 and 60 min trenH-SnS-1 (see Figs. S1b and S6). PDFs of pristine and water-treated (*c*) AEPz-SnS-1 and (*d*) trenH-SnS-1.

The PXRD data are shown in Figs. 2 ▸(*a*) and 2(*b*), and the X-ray total scattering data collected on the same samples are shown in Figs. S3 and S4 for the range *Q* = 0.5–19.5 Å^−1^. For both water-treated samples, a gradual shift of the (002) reflection to higher scattering vector *Q* is observed with increasing time in water compared with the pristine sample [Figs. 2 ▸(*a*) and 2(*b*)]. This shift is indicative of the formation of shorter average interlayer distances. The evolution of the interlayer distance based on the position of the (002) reflection is shown in Fig. 5(*a*) (see later discussion). For comparison, the position of the (100) reflection largely remains unchanged at 0.55 Å^−1^, hinting that the intralayer structure is partly conserved. PDF was performed to obtain better insight into the structural transformation.

### 3.2. Short-range order (<8 Å)   

**Figure 3:**
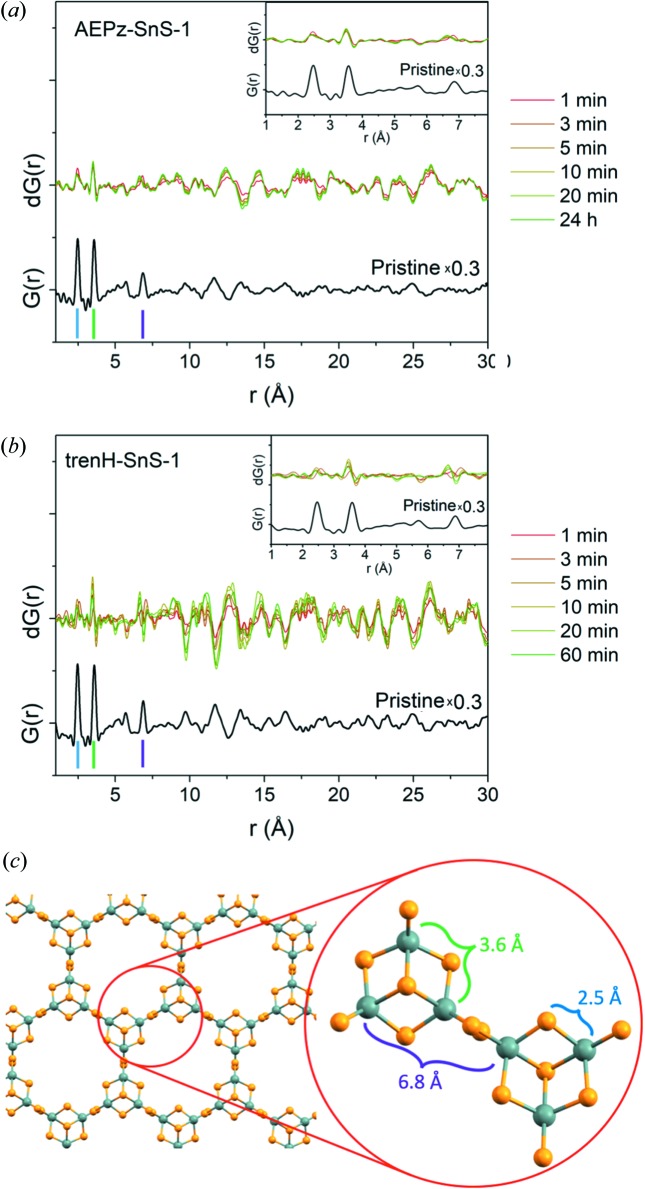
Differential PDFs [d*G*(*r*) = PDF_*t*_ − PDF_*t* = 0_] of (*a*) AEPz-SnS-1 and (*b*) trenH-SnS-1 at different times *t* of dispersion in water. The PDFs of the pristine compounds (scaled ×0.3) are shown (black) below the dPDFs for comparison. Insets: dPDFs of short-range correlations. (*c*) Thio­stannate layer, highlighting two clusters and interatomic distances of pronounced correlations in the PDFs.

PDFs based on the total scattering data are displayed in Figs. 2 ▸(*c*), 2(*d*) and S5. At first glance, it is seen that the dominant correlations are preserved at short distances (<8 Å), *i.e.* not taking interlayer correlations into account. In order to investigate minor structural changes, differential PDFs (dPDFs) were obtained by subtracting the PDF of the pristine compounds from those of the water-treated samples [Figs. 3 ▸(*a*), 3(*b*) and S7]. Hence, positive peaks in the dPDFs indicate the formation of new interatomic correlations during water-exposure, whereas negative peaks correspond to interatomic distances in the pristine compound that vanish in the treated samples. Each short-range peak individually corresponds to a single correlation in the structure, as seen in Fig. 3 ▸(*c*).

Not surprisingly, the most intense short-range order features in the PDFs are the Sn—S bonds at 2.5 Å and the shortest Sn—Sn distances at 3.6 Å. Note that the peaks at 0.8 Å [Figs. 2 ▸(*c*) and 2(*d*)] are not physical, but are a result of the Fourier transformation, and they should therefore not be treated as an interatomic correlation, whereas the minor peaks at 1.2, 1.5 and 2.0 Å all correlate to interatomic distances in the organic cations. The peak at 6.8 Å can be assigned to Sn—Sn distances between Sn atoms located in neighboring clusters [Fig. 3 ▸(*c*)]. Correlations above 2 Å relating to the cations AEPz and trenH are not observed in the PDFs, given their low scattering power in comparison with Sn and S. Thus, effectively only changes to the [Sn_3_S_7_^2−^]*_n_* structure are observable in the PDFs. For both samples, the peaks in the short-range section (<8 Å) of the PDFs largely remain unchanged throughout the water treatment, as only the Sn—Sn correlations at 3.6 Å shift to slightly shorter distances, especially in trenH-SnS-1 [Figs. 3 ▸(*b*) and S7]. This development confirms the preservation of the Sn—S bonds and the broken-cube clusters in the process. Furthermore, as no new peaks are formed in this region, the amorphization is probably not associated with extensive formation of new bonds. Instead, the loss of crystallinity may involve, for example, layer exfoliation, rotational disorder between the layers, or packing of the layers in a non-parallel manner. Stacking faults would result in any of these cases, and such structural changes would be consistent with the preserved broken-cube clusters.

### 3.3. Intermediate-range order (8–30 Å)   

Peaks at higher *r*-values (>8 Å) are subject to noticeable changes during the progression of the water treatment [Figs. 3 ▸(*a*) and 3(*b*)]. Several interatomic distances correspond to new as well as vanishing correlations. Simple peak assignment is not trivial as numerous correlations, intralayer as well as interlayer, are present at 8–30 Å. The structural changes at *r* > 8 Å are more pronounced for trenH-SnS-1 than for AEPz-SnS-1. This illustrates that while the thio­stannate layers of the two compounds are isostructural, the difference between the amine-based cations has a significant impact on the stability of the materials in water suspension. As such, it is possible that the amorphization involves an acid–base reaction, *i.e.* deprotonation of trenH^+^ or AEPzH^+^ cations by the water molecules to yield neutral tren or AEPz molecules, and that charge balance in the solid is maintained by small H_3_O^+^ cations. Obviously, the electrostatic interactions between the cations and the thio­stannate layers would then be altered. The deprotonation step would depend on the amine basicity, although we also suspect the bulkiness and electrostatic interactions (leading to order/disorder) with the thio­stannate framework to be important in the decomposition. For example, the water-stable compound Sn_3_S_7_(DABCOH)_2_ (Jiang *et al.*, 1998*c* ▸) contains crystallographically ordered cations, but the p*K*_a_ of DABCOH^+^ (8.8) is comparable to that of AEPzH^+^ (assumed to be similar to that of 1-methyl­piperazineH^+^ of 9.14) and trenH^+^ (assumed to be similar to that of di­ethyl­entri­amineH^+^ of 10.45). Combined effects of amine basicity and electrostatic interactions may explain the observed difference in stability of trenH-SnS-1 and AEPz-SnS-1. The proposed acid–base mechanism is further supported by pH measurements of concentrated suspensions of the two *R*-SnS-1 compounds (~430 mg trenH-SnS-1 or AEPz-SnS-1, in 10 ml). The pH in the aqueous solution increased by 2.3 units for AEPz-SnS-1 and 2.5 units for trenH-SnS-1 over 24 h as the thio­stannates are amorphisized, which is likely to be a result of the liberation of the basic amines into solution. The ^1^H NMR spectrum of the amines leaching into solution (D_2_O) from trenH-SnS-1 (Figs. S12 and S13) revealed several signals expected for aliphatic amines. However, many more peaks were found than can be assigned to tren, trenH^+^ or any simple molecular species. It is likely that the observed complexity of the NMR spectrum stems from degradation products of tren, which were formed during the solvothermal synthesis. A completely different observation suggesting that the amorphization is driven by an acid–base reaction involving water and the amines is the fact that both compounds remain crystalline in ethanol (Fig. S2).

### 3.4. Domain size and interlayer distance   

The PDF data were refined in real space using the *PDFfit2* code and *PDFgui* interface (Farrow *et al.*, 2007 ▸) with the aim of obtaining additional structural information. The applied structural model has some limitations as it is based on: (i) the hexagonal structure of the pristine thio­stannate (*P*6_3_/*mmc*), which is not maintained, and (ii) a spherical domain size even though two-dimensional materials are anisotropic, as covalent bonds dominate in-plane, while weaker electrostatic and Van der Waals interactions hold together the layers. Nevertheless, reasonable fits to the PDFs were obtained, and the refined parameters have been summarized in Tables S1–S3, and Figs. S8 and S9 display examples of data fits. In the following, unless otherwise stated, the atomic displacement parameters of both *R*-SnS-1 data series have been refined isotropically.

**Figure 4:**
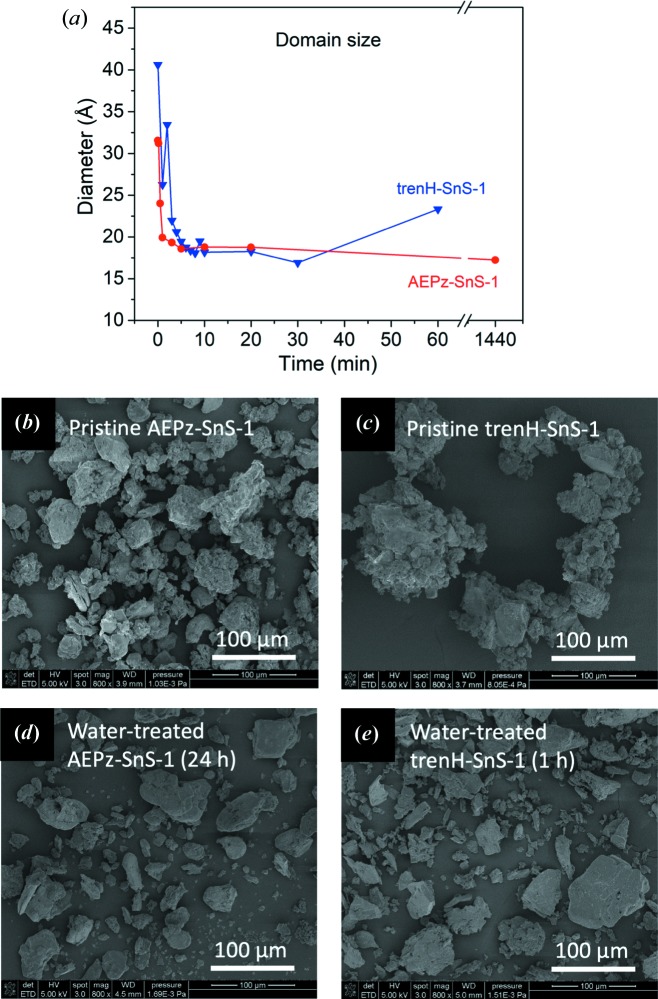
(*a*) Spherical domain size obtained by refinement of tren-SnS-1 and AEPz-SnS-1 PDF data displayed as a function of time in water. The lines are guides to the eyes. SEM images of (*b*) pristine AEPz-SnS-1, (*c*) pristine trenH-SnS-1, (*d*) water-treated AEPz-SnS-1 (24 h) and (*e*) water-treated trenH-SnS-1 (1 h).

The spherical domain sizes of AEPz-SnS-1 and trenH-SnS-1 were extracted from the PDF refinements [Fig. 4 ▸(*a*)]. Clearly, the domain size of the samples rapidly diminishes over time, reaching equilibrium values after about 10 min, and the similarity between the two compounds is striking. A decrease in domain size is expected for an amorphization process, as the crystal structure becomes insufficient to describe the compound beyond the local structure. However, it should be noted that the absolute values of the coherent scattering domain sizes do not represent the physical particle sizes, as they were determined to be 32 (3) and 41 (3) Å for pristine AEPz-SnS-1 and trenH-SnS-1, respectively. These domain sizes are much smaller than the micrometre-sized pristine crystals obtained from the solvothermal synthesis according to: the sharp X-ray diffraction lines [Figs. 2 ▸(*a*) and 2(*b*)] and SEM images [Fig. 4 ▸(*b*) and 4(*c*)]. Likewise, the PDF sizes of the water-treated particles are also underestimated, as the crystalline model poorly describes correlations at distances larger than approximately 15 Å (Fig. S9). After water treatment, smaller particles (∼200–400 nm) are also formed (Fig. S14) in addition to micrometre-sized particles [Figs. 4 ▸(*d*) and 4(*e*)]. Instead of the absolute size, we interpret only on the trend: the crystalline domain size decreases with time spent in the water suspension.

To study the effects of mechanical force on the amorphization of the *R*-SnS-1 compounds, we applied magnetic stirring to a solution containing trenH-SnS-1 for 24 h. In Fig. S10a, the total scattering pattern of the resulting product is compared with that of a non-stirred sample (24 h). The patterns show a clear difference in crystallinity between the samples: when no stirring is applied, the thio­stannate framework is altered but still retains some characteristic peaks. When stirring is applied, almost all characteristic peaks are lost. Thus, we conclude that while water treatment without stirring causes amorphization of the structure, mechanical force causes a more severe alteration of the framework with significant loss of crystallinity when the dispersion time is kept constant. Nevertheless, both samples retain the local structure of the trenH-SnS-1 framework, as evident from the low-*r* region of their PDFs (Fig. S10b). Based on this, we propose that although a decreased particle size along the stacking direction can be achieved by delamination of the layered structure, decreasing the in-plane dimensions would require breaking of strong covalent Sn—S bonds. Such bond breaking might occur if the thio­stannate particles become sufficiently thin and mechanical stress causes the layers to break into smaller fragments.

**Figure 5:**
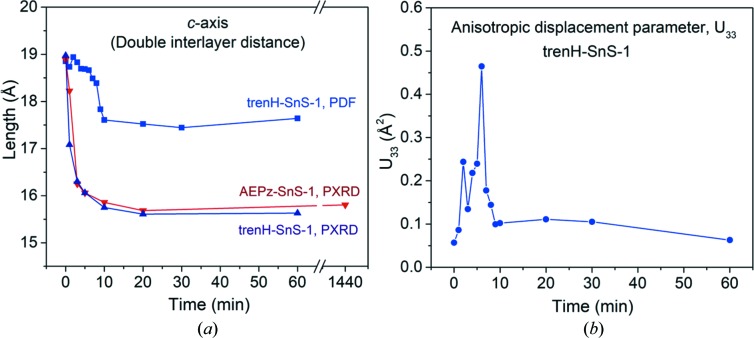
(*a*) The double interplanar distance (corresponding to the crystallographic *c* axis) as a function of time in water as obtained from PXRD (AEPz-SnS-1 and trenH-SnS-1) and PDF (trenH-SnS-1). (*b*) The displacement parameter *U*_33_ (*i.e.* perpendicular to the [Sn_3_S_7_^2−^]*_n_* plane) of the tin atoms using an anisotropic trenH-SnS-1 PDF model (Table S3). The lines are guides to the eyes.

The evolution of the crystallographic *c* axis (corresponding to the double interlayer distance) over time as determined from the PXRD patterns and PDF refinements is presented in Fig. 5 ▸(*a*). The PXRD values were simply estimated from the center of the shifting (002) reflection in the diffraction patterns [Figs. 2 ▸(*a*) and 2(*b*)]. Based on the PXRD (002) reflection, the *c* axis is found to decrease from 19.0 to 15.8 Å for AEPz-SnS-1, and from 19.1 to 15.5 Å for trenH-SnS-1 with the largest changes occurring within the first 10 min of water treatment. The change in interlayer spacing translates to 1.6 Å for AEPz-SnS-1 and 1.8 Å for trenH-SnS-1. However, given the width of the (002) peaks, the interlayer spacing is probably very non-uniform and presumably represents a distribution of distances, non-parallel layer packing and/or stacking faults. Asymmetric tailing of (00*l*) reflections may be indicative of turbostratic disorder (Grangeon *et al.*, 2013 ▸), and is observable in several of the diffraction patterns.

PDF refinements of the trenH-SnS-1 data series also revealed a decreasing trend in the crystallographic *c* axis, although to a lesser extent, with the *c* axis dimension settling at 17.6 Å [Fig. 5 ▸(*a*)]. No such trend was found in the PDF refinements of the AEPz-SnS-1 data, possibly because the less pronounced changes to AEPz-SnS-1 carried insufficient information about the decrease in the interlayer distance [compare Figs. 3 ▸(*a*) and 3 ▸(*b*)]. It should be noted that the interplanar distances obtained from PXRD are likely to be more reliable than those obtained by PDF, as they can be extracted directly from the diffraction patterns, whereas those obtained from PDF refinements are model dependent.

A second refinement of the trenH-SnS-1 data was performed using anisotropic atomic displacement parameters (*U*_11_ = *U*_22_ ≠ *U*_33_). This produced the same decreasing trend of interplanar distance (Table S3) as found for the isotropic model. Furthermore, the anisotropic *U*_33_ displacement parameter of the Sn atoms perpendicular to the thio­stannate layers increases substantially within the first 10 min, as shown in Fig. 5 ▸(*b*). From its initial value of 0.057 Å^2^ for the pristine sample, *U*_33_ increases to more than 0.2 Å^2^ for several points during the 2–10 min interval, peaking at 0.46 Å^2^ after water treatment for 6 min. These displacement parameters are unphysically large, suggesting significant static positional disorder along the stacking direction, *i.e.* the thio­stannate sheets are not separated by a fixed interlayer distance. Fixing the *c* axis in the PDF refinements to the values obtained from PXRD also produced unphysically large Sn displacement parameters along the stacking direction, *cf*. Table S4. Overall, both PDF refinements and PXRD provide evidence of a non-uniform decrease in interlayer spacing in the [Sn_3_S_7_^2−^]*_n_* frameworks, as much as 1.8 Å.

### 3.5. Intralayer and interlayer peak assignment   

**Figure 6:**
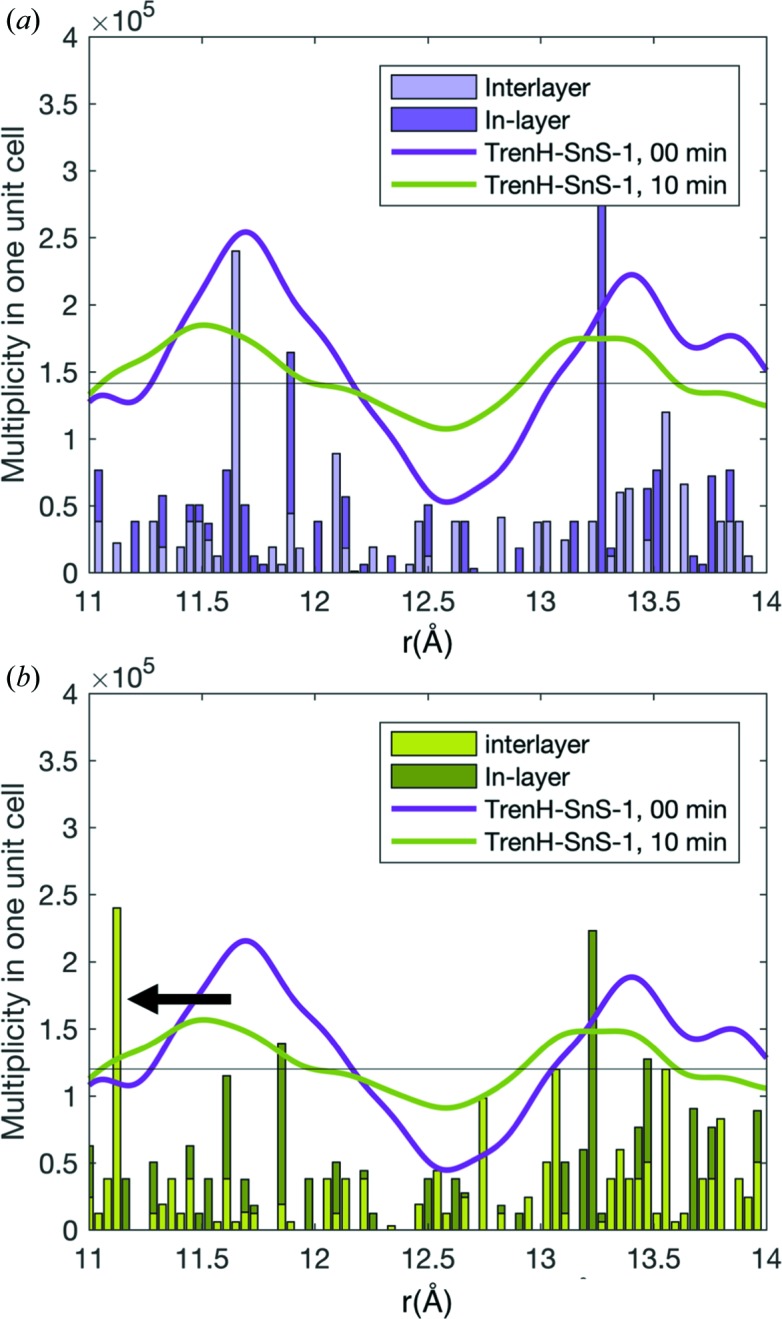
Solid lines are the experimental PDF data of pristine (purple) and water-treated (green) trenH-SnS-1 [same data in (*a*) and (*b*)]. Histograms representing pair correlations (11–14 Å) resulting from the refinement of the PDFs of trenH-SnS-1 in water at (*a*) 0 min (pristine) and (*b*) 10 min. The histograms represent the number of interatomic distances (weighted by the scattering power of the atoms in the pair) according to the refined models. The correlations have been divided into intralayer/in-layer (dark purple or dark green) and interlayer (light purple or light green) correlations. The apparent downwards shift of the strong scattering interlayer correlations at 11.7 Å in (*a*) to 11.2 Å in (*b*) are highlighted with an arrow.

In order to examine if the largest water-induced structural changes are observed for correlations *within* one plane (intralayer/in-layer) or *between* different planes (interlayer), the output of the PDF refinements in *PDFGui* was examined further. As the most pronounced structural changes were observed for trenH-SnS-1 [Figs. 3 ▸(*a*) and 3(*b*)], all interatomic correlations (<14.5 Å) were extracted from the fits to pristine and water-treated (10 min) PDF data of this compound. The pair correlations were intensity weighted by their atomic scattering power and sorted into interlayer and intralayer correlations using a MATLAB script. Subsequently, the correlations were plotted in a histogram. A detailed description of the pair extraction and weighting scheme is given in the supporting information. A section of the histogram (11–14 Å) is shown for trenH-SnS-1 at 0 min [Figs. 6 ▸(*a*)] and 10 min [Fig. 6 ▸(*b*)] along with the experimental data forming the basis of the refinements.

For the pristine compound [Fig. 6 ▸(*a*)], a good agreement between the histogram (based on fitted parameters) and experimental data (purple line) is observed. For example, the model describes a strong interlayer correlation at 11.7 Å, as indicated by the tall light-purple bar, which coincides with a distinct peak in the data. This validates the quality of the PDF refinement. For the 10 min data, this strong interlayer correlation has shifted to 11.2 Å [Fig. 6 ▸(*b*)]. However, the data show no clear peak at this position, *i.e.* the model vastly overestimates the intensity at 11.2 Å, as is also noticeable from the obtained fit (Fig S9). This shows that substantial structural changes can no longer be described by the crystalline model; in particular, the interlayer correlations are poorly described by the model. As concluded from the PXRD studies, the amorphization of the *R*-SnS-1 compounds is likely to be associated with some degree of compaction and non-uniform stacking of the [Sn_3_S_7_^2−^]*_n_* layers, which smear out the PDF peaks related to interlayer correlations and therefore do not produce distinct peaks. This explanation is further supported by the broad (002) PXRD peaks and unphysically large displacement parameters of the Sn atoms perpendicular to the thio­stannate planes [anisotropic model, Fig. 5 ▸(*b*), Table S3] as previously discussed.

In an attempt to extract additional information from the PDF data, anisotropic and crystal size dependent Debye refinements were performed from single and double sheets of the structure, and theoretical PDFs were calculated using *Diffpy-CMI* (Juhás *et al.*, 2015 ▸) (see the supporting information). These methods did not conclusively result in additional structural information.

## 4. Chemical composition   

The decrease in interlayer spacing is associated with a decrease in the organic cation concentration as shown by XPS, elemental analysis and pH measurements. The elemental composition of the surface was determined from high-resolution XPS spectra. Due to the sample stoichiometry of [Sn_3_S_7_^2−^]*_n_*, all concentrations are presented relative to Sn = 3. The C and N concentrations of both AEPz-SnS-1 and trenH-SnS-1 decreased by the same ratio of approximately 2–3 after water exposure (Table 1 ▸). The reduced C and N content is indicative of the removal of amines from the sample, which is in agreement with the partial structural collapse. The sulfur peaks have been divided into two components, *i.e.* one matching sulfide in *R*-SnS-1 (161.5 eV), whereas the other is a minor component of SO_*x*_ (possibly sulfate) at 167.9 eV. After soaking in water, both samples show a very low content of SO_*x*_, which indicates the sulfur impurity has largely been removed. Clearly, an Sn:S ratio of 3:7 is not observed for the pristine samples, which may be explained by a different surface structure of the *R*-SnS-1 compounds. In addition, the C/N ratio is higher than that of the intercalated molecules (*i.e.* C/N is formally 2 for AEPz and 3/2 for tren). The carbon excess is expected to arise from adventitious carbon on the sample surface. Therefore, the amine content should only be considered as a relative rather than absolute value. Spectra and atomic percentages of the four samples are shown in Figs. S15–S18 and Tables S5–S9 of the supporting information.

Using elemental analysis, the trend observed from surface-sensitive XPS was confirmed to be a reduction of the organic parts compared with sulfur. Both bulk samples of AEPz-SnS-1 and trenH-SnS-1 have reduced the organic concentrations by approximately 30% compared with the sulfur content (Tables 2 ▸ and S10). The small differences between the calculated atomic stoichiometry and the pristine samples are expected to originate from residual amines left on the surface. Notably, the atomic ratio of C/N and C/H is seen to remain constant for samples both before and after treatment and in all cases match the expected ratio of the amines. Meanwhile, the C/S ratio is seen to decrease after the treatment in both cases, hence supporting the hypothesis of cation removal.

Based on PXRD, total scattering, electron microscopy and XPS data, we propose the following mechanism for the amorphization: water diffuses into crystalline *R*-SnS-1 and acts as a base by partly deprotonating the cations residing between the thio­stannate layers. This causes changes in the hydrogen bonding/electrostatic interactions holding the layers together, and the deprotonated amines partly diffuse out of the solid and into the solution. This process results in highly disordered [Sn_3_S_7_^2−^]*_n_* layers in partially delaminated structures associated with an average decrease in the interlayer distance. The delamination results in fragile layers, which are cleaved to smaller domains induced by mechanical stress, as demonstrated in the stirred samples. Yet it remains unknown if the charge balance is maintained by protons binding to the exfoliated thio­stannate sheets, H_3_O^+^ residing between the layers, or whether defects leading to charge balance are introduced in the process. The pristine compounds are stable in moist air and following the addition of small quantities of water, suggesting that water in excess is needed to shift the acid/base equilibrium. Differences in the p*K_a_* values of AEPzH^+^ and trenH^+^ and their electrostatic interactions may explain the different stabilities of AEPz-SnS-1 and trenH-SnS-1 in water.

## 5. Optical properties   

**Figure 7:**
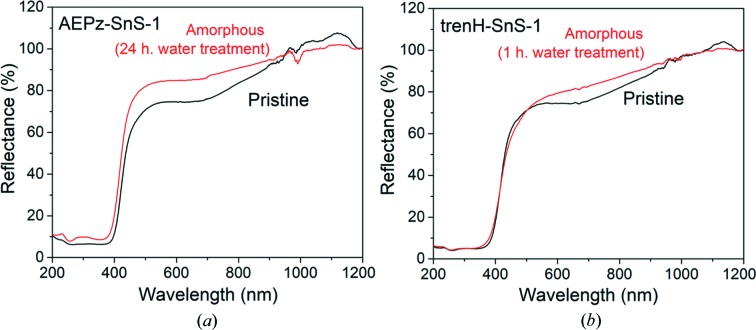
Diffuse reflectance spectra of pristine and amorphous (*a*) AEPz-SnS-1 and (*b*) trenH-SnS-1.

As the local structure of the thio­stannate framework remains intact, it is worth examining if some of the properties of the pristine compounds persist in the amorphous products. The light absorption properties of pristine and amorphous AEPz-SnS-1 and trenH-SnS-1 were investigated by DRS. As shown in Fig. 7 ▸, only small differences in light absorption are observed between the pristine and amorphous samples. Absorption edges are observed around 400 nm for both crystalline and amorphous AEPz-SnS-1 and trenH-SnS-1, and converting the reflectance data by the Kubelka–Munk function (Fig. S19) yields band gaps of 2.95 eV (AEPz-SnS-1, pristine), 2.99 eV (AEPz-SnS-1, 24 h in H_2_O), 3.02 eV (trenH-SnS-1, pristine) and 3.00 eV (trenH-SnS-1, 1 h in H_2_O). The underlying electronic transitions of the thio­stannates therefore appear to be unaffected by the change from crystalline to amorphous phases, and the compounds remain semiconducting despite the structural alterations. This indicates that the electronic transition from the valence band to the conduction band is mainly localized within one layer. The preserved optical properties are highly encouraging for the application of *R*-SnS-1 compounds in aqueous solution.

## 6. Conclusions   

The water-mediated amorphization of two layered thio­stannates, AEPz-SnS-1 and trenH-SnS-1, has been studied in detail. To obtain information about the structural progression, we conducted a PDF analysis of the X-ray total scattering data of the *R*-SnS-1 samples dispersed in water for different durations. As evident from these in-depth analyses, the transition from crystalline to amorphous does not involve complete decomposition of the thio­stannate framework, as the local structure is preserved. Data modeling suggests a rapid decrease in the crystalline domain sizes, reaching an equilibrium for both compounds within 10 min of water exposure. Analysis of the (002) Bragg peak position of the PXRD data revealed a substantial decrease in the average interlayer distance by more than 1.5 Å for both compounds. The amorphization is associated with partial delamination and disordered (rotational and non-parallel) stacking of the layers, which is probably related to partial removal of the organic components from within the framework. The thio­stannate layers can also be broken into smaller fragments if the thinner particles become susceptible to the stress of mechanical force. The light absorption properties of AEPz-SnS-1 and trenH-SnS-1 are not affected by the structural transition, while increasing the surface area of the material through the formation of smaller particles. Hence, the water treatment is a sustainable route for the modification of such materials with promising catalytic applications.

## 7. Related literature   

The following references have been cited in the supporting information: Baur & Kahn (1971 ▸); Moulder & Chastain (1992 ▸).

## Supplementary Material

Supporting figures and data. DOI: 10.1107/S2052252519006791/yu5016sup1.pdf

## Figures and Tables

**Table 1:** Relative atomic concentrations as determined from high-resolution XPS spectra The numbers are shown as referenced values, where atom ratios have been calculated relative to a fixed value of Sn = 3.0. The theoretical values are based on a pristine sample with no contaminants and with two organic amines per (Sn_3_S_7_^2−^)_n_.

XPS	AEPz-SnS-1 theoretical	AEPz-SnS-1 pristine	AEPz-SnS-1 24 h	TrenH-SnS-1 theoretical	trenH-SnS-1 pristine	trenH-SnS-1 1 h
C	12	31.5 ± 0.8	15.5 ± 0.5	12	38.7 ± 1.0	16.1 ± 1.8
N	6	11.0 ± 0.3	4.4 ± 0.2	8	15.2 ± 0.6	3.8 ± 0.04
O	–	9.0 ± 0.8	3.4 ± 0.1	–	11.2 ± 0.6	5.1 ± 0.2
S	7	4.0 ± 0.1	3.9 ± 0.1	7	4.0 ± 0.1	3.6 ± 0.1
SO_*x*_	–	0.7 ± 0.1	0.2 ± 0.05	–	0.9 ± 0.03	0.2 ± 0.04
Sn	3	3.0	3.0	3	3.0	3.0

**Table 2:** Elemental analysis of AEPz-SnS-1 and trenH-SnS-1, both pristine and water-treated for 1 h All atomic stoichiometries have been referenced to seven sulfur atoms. Expected values are based on a pristine sample with no contaminants and with two monoprotonated organic amines per (Sn_3_S_7_^2−^)_*n*_.

Atoms	AEPz-SnS-1 theoretical	AEPz-SnS-1 pristine	AEPz-SnS-1 24 h	TrenH-SnS-1 theoretical	TrenH-SnS-1 pristine	TrenH-SnS-1 1 h
C	12	15.08 ± 0.04	9.81 ± 0.03	12	13.62 ± 0.08	8.27 ± 0.13
H	32	38.13 ± 2.03	16.44 ± 1.56	38	36.48 ± 2.02	24.01 ± 1.23
N	6	7.29 ± 0.01	4.79 ± 0.02	8	7.03 ± 0.05	4.49 ± 0.08
S	7	7	7	7	7	7
C/N	2.00	2.07	2.05	1.5	1.94	1.84
C/H	0.38	0.40	0.37	0.32	0.37	0.34
C/S	1.71	2.15	1.40	1.71	1.95	1.18
