# Supplementary material for: Structural changes during water-mediated amorphization of semiconducting two-dimensional thio­stannates
Source: IUCrJ. 2019 Jul 5;6(Pt 5):804–14. doi: 10.1107/S2052252519006791 (PMC6760444; doi:10.1107/S2052252519006791)
Supplement: Supplementary file 1 [file m-06-00804-sup1.pdf]

IUCrJ

Volume 6 (2019)

Supporting information for article:

Structural changes during water-mediated amorphization of semiconducting two-dimensional thiostannates

Mathias S. Hvid, Henrik S. Jeppesen, Matteo Miola, Paolo Lamagni, Ren Su, Kirsten M. Ø. Jensen and Nina Lock

Supporting information

Structural changes during water-mediated amorphization of semiconducting two-dimensional thiostannates

Mathias S. Hvid,[a§] Henrik S. Jeppesen,[b§] Matteo Miola,[c] Paolo Lamagni,[c] Ren Su[d], Kirsten M. Ø. Jensen,[e] and Nina Lock[f]*

[a]Interdisciplinary Nanoscience Center (iNANO), Aarhus University, Gustav Wieds Vej 14, DK-8000 Aarhus C, Denmark

[b]Sino-Danish Center for Education and Research (SDC), Interdisciplinary Nanoscience Center (iNANO), Aarhus University, Gustav Wieds Vej 14, DK-8000 Aarhus C, Denmark

[c]Carbon Dioxide Activations Center (CADIAC), Interdisciplinary Nanoscience Center (iNANO), Aarhus University, Gustav Wieds Vej 14, DK-8000 Aarhus C, Denmark

[d]SynCat@Beijing, Synfuels China Technology Co. Ltd., Leyuan South Street II, No.1, Yanqi Economic Development Zone C#, Huairou District, Beijing, 101407, China.

[e]Dept. of Chemistry, University of Copenhagen, Universitetsparken 5, 2100 København Ø

[f]Carbon Dioxide Activation Center, Interdisciplinary Nanoscience Center (iNANO) and Dept. of Chemistry, Aarhus University, Gustav Wieds Vej 14, DK-8000 Aarhus C, Denmark

(current address: Dept. of Engineering, Aarhus University, Åbogade 40, DK-8200 Aarhus N, Denmark)

[§]These authors contributed equally to the study

*Corresponding author: nlock@eng.au.dk

1. PXRD data

1.1 Le Bail fits of pristine AEPz-SnS-1 and trenH-SnS-1

The result of Le Bail refinements of PXRD data collected on pristine samples of AEPz-SnS-1 (Fig S1a) and trenH-SnS-1 (Fig. S1b).

AEPz-SnS-1: $a=b=13.3037(3)$ Å, $c=19.2195(5)$ Å, $R_F=0.24$.
trenH-SnS-1: $a=b=13.2457(4)$ Å, $c=19.0860(8)$ Å, $R_F=0.10$.

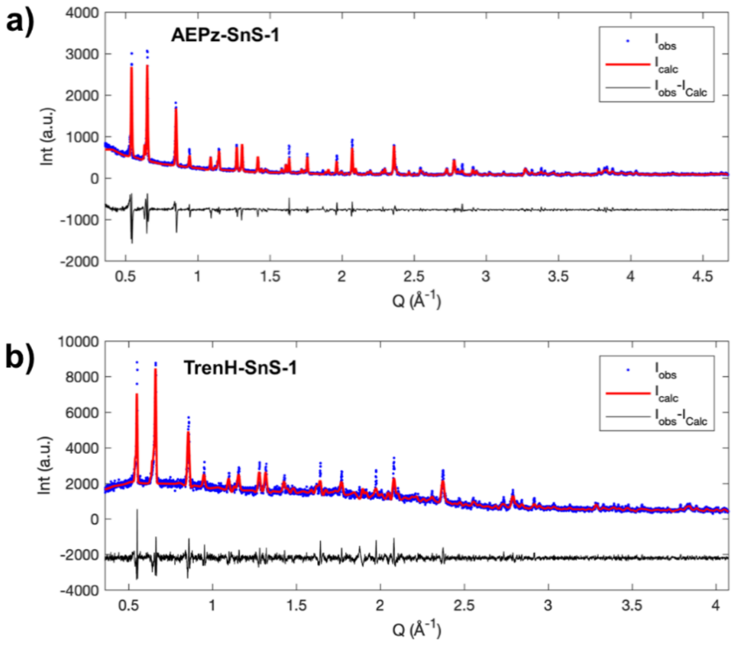

Figure S1: Le Bail refinement of pristine a) AEPz-SnS-1 and b) trenH-SnS-1.

1.2 PXRD data of AEPz-SnS-1 and trenH-SnS-1 dispersed in ethanol

PXRD patterns of samples dispersed in ethanol for 1 h were collected on a STOE STADI P diffractometer using Cu$K_{\alpha1}$ radiation (1.54056Å) at room temperature. Ground powder samples were distributed on a piece of sticky tape, and data were collected in transmission geometry.

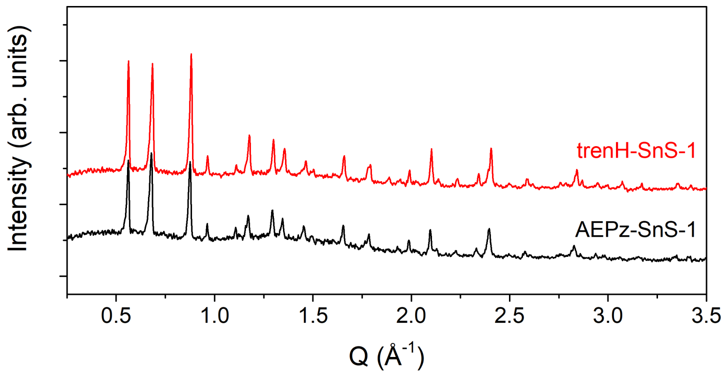

Figure S2: PXRD patterns of AEPz-SnS-1 and trenH-SnS-1 samples dispersed in ethanol for 1 h, no stirring. The samples remain crystalline in ethanol in contrast to water.

2. Synchrotron total scattering data of water-mediated samples

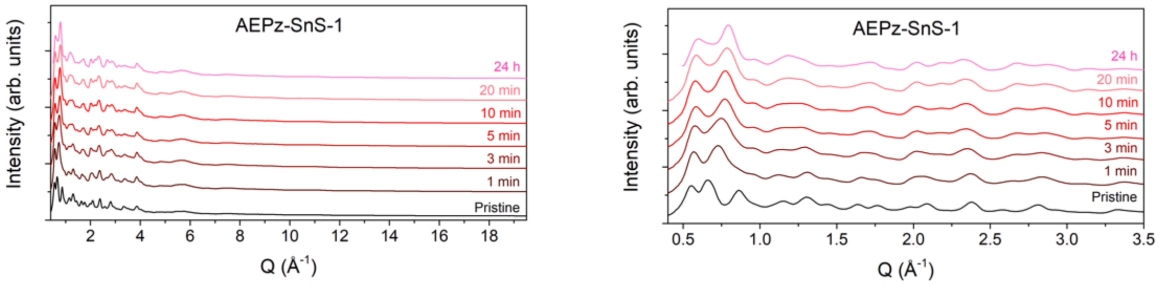

Figure S3: Synchrotron total scattering data of pristine and water-treated AEPz-SnS-1, all data (left) and low Q (right).

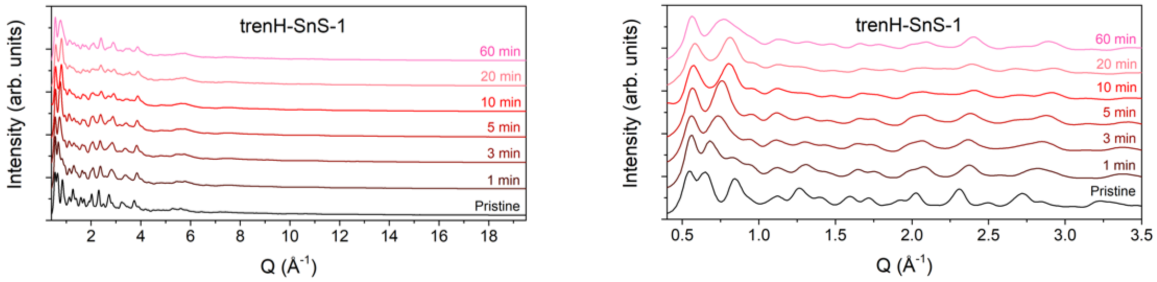

Figure S4: Synchrotron total scattering data of pristine and water-treated trenH-SnS-1, all data (left) and low Q (right).

3. Pair distribution functions of water-mediated samples

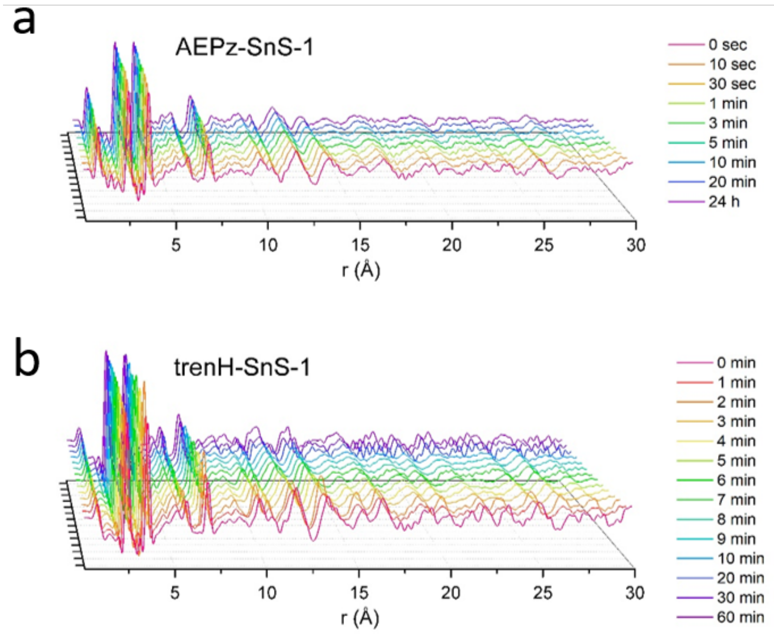

Figure S5: PDFs of pristine and water-treated a) AEPz-SnS-1 and b) trenH-SnS-1.

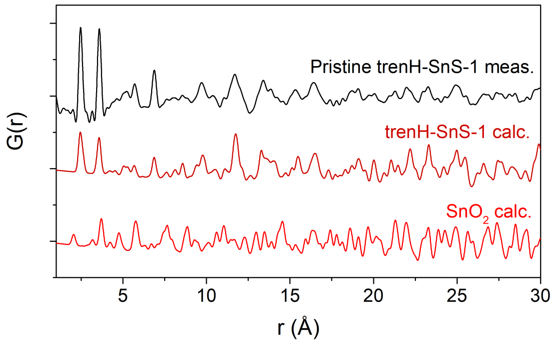

Figure S6: Calculated trenH-SnS-1 and SnO_2 PDFs based on reported crystal structures (Baur & Khan, 1971, Filsø et al., 2017), compared with the experimental PDF of trenH-SnS-1.

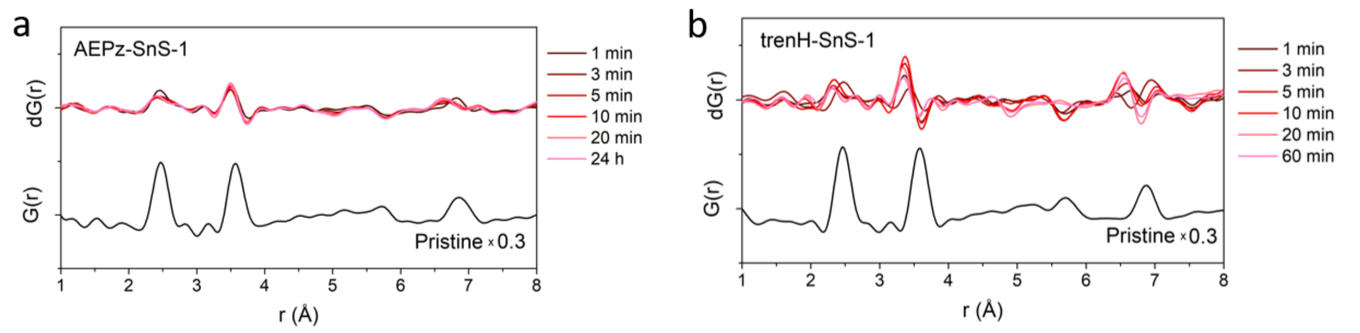

Figure S7: Differential PDFs ($dG(r) = PDF_t - PDF_{t=0}$) of a) AEPz-SnS-1 and b) trenH-SnS-1 at different durations t of dispersion in water. The PDFs of the pristine compounds (scaled x0.3) are shown (black) below the dPDFs for comparison. dPDFs in the range 1-30 Å are shown in the manuscript Fig. 3.

4. PDF refinements

4.1 PDF fits

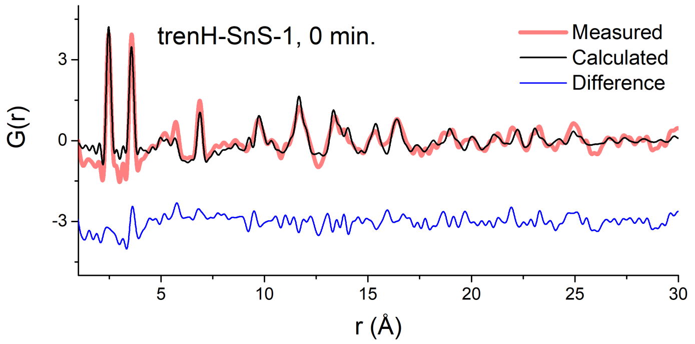

Figure S8: PDF data fit of pristine trenH-SnS-1, isotropic ADPs.

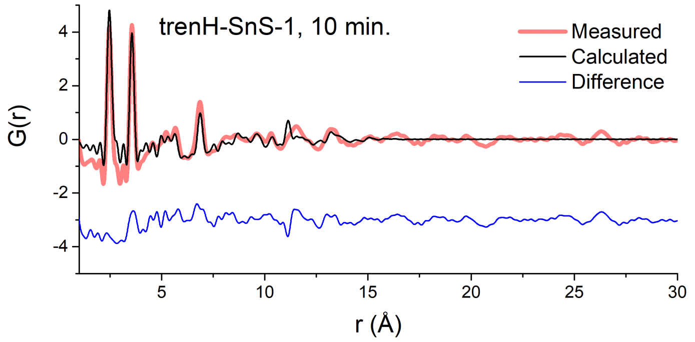

Figure S9: PDF data fit of trenH-SnS-1 after 10 min. in H_2O, isotropic ADPs.

The isostructural thiostannate layers in AEPz-SnS-1 and trenH-SnS-1 has $P6_3/mmc$ symmetry and contains four atoms in the asymmetric unit. Refinement details are shown in Table S1-S3.

4.2 AEPz-SnS-1, isotropic atomic displacement model

Time (min)	Scale factor	a (Å)	c (Å)	Delta2	SP Dia (Å)	U_{iso}(Sn) (Å^2)	U_{iso}(S) (Å^2)
**0**	0.79(6)	13.262(25)	18.965(65)	5.3(1.2)	31.5(3.1)	0.0050(12)	0.0137(5)
**0.167**	0.84(8)	13.260(24)	18.773(77)	5.96(43)	31.2(3.3)	0.0035(8)	0.0144(6)
**0.5**	1.0(1)	13.248(25)	18.924(75)	5.81(15)	24.0(2.0)	0.0046(10)	0.0180(7)
**1**	1.15(9)	13.266(25)	18.810(83)	5.6(1.0)	19.9(1.4)	0.0039(9)	0.0205(8)
**3**	0.97(8)	13.265(27)	18.816(84)	5.3(1.1)	19.3(1.5)	0.0029(9)	0.0142(7)
**5**	0.96(8)	13.264(28)	18.811(92)	5.6(1.1)	18.6(1.5)	0.0029(9)	0.0144(8)
**10**	0.97(8)	13.264(27)	18.810(87)	5.6(1.1)	18.8(1.5)	0.0028(9)	0.0143(7)
**20**	0.97(8)	13.263(27)	18.810(87)	5.6(1.1)	18.8(1.5)	0.0028(9)	0.0144(7)
**1440**	1.03(8)	13.258(29)	18.78(11)	5.5(1.9)	17.2(1.4)	0.0029(11)	0.0117(7)

Time (min)	Sn x	Sn z	S1 z	S2 x	S2 z	S3 x	S3 z	Red Chi sq	Rw
**0**	0.4239(5)	0.494(1)	0.554(6)	0.773(3)	0.438(4)	0.489(2)	0.594(3)	0.04918	0.47895
**0.167**	0.4232(7)	0.494(1)	0.56(1)	0.773(4)	0.436(5)	0.489(2)	0.594(4)	0.03793	0.40752
**0.5**	0.4232(7)	0.494(1)	0.56(1)	0.772(4)	0.440(5)	0.489(1)	0.593(2)	0.03984	0.40760
**1**	0.4232(6)	0.496(1)	0.561(6)	0.772(4)	0.441(6)	0.489(2)	0.593(3)	0.03514	0.38899
**3**	0.4231(7)	0.495(2)	0.561(8)	0.770(4)	0.434(5)	0.490(2)	0.594(3)	0.03499	0.40437
**5**	0.4232(7)	0.496(2)	0.562(7)	0.770(4)	0.433(5)	0.489(2)	0.593(3)	0.03524	0.41137
**10**	0.4232(7)	0.496(2)	0.562(7)	0.770(4)	0.433(5)	0.489(2)	0.593(3)	0.03733	0.41596
**20**	0.4231(7)	0.496(2)	0.562(7)	0.770(4)	0.433(5)	0.489(2)	0.593(3)	0.03904	0.42132
**1440**	0.4233(7)	0.495(2)	0.583(4)	0.770(4)	0.439(6)	0.491(2)	0.589(3)	0.03696	0.41552

Table S1: Refinement parameters of AEPz-SnS-1 fits using isotropic atomic displacement parameters.

4.3 trenH-SnS-1, isotropic atomic displacement model

Time (min)	Scale factor	a (Å)	c (Å)	Delta2	SP Dia (Å)	U_{iso}(Sn) (Å^2)	U_{iso}(S) (Å^2)
**0**	1.10(3)	13.298(13)	18.852(38)	5.79(49)	40.6(3.0)	0.0054(8)	0.0135(7)
**1**	1.26(8)	13.275(16)	18.735(53)	5.82(38)	26.3(1.6)	0.0045(10)	0.0119(8)
**2**	1.20(7)	13.345(12)	18.939(38)	5.91(16)	33.4(2.1)	0.0038(7)	0.0109(6)
**3**	1.37(10)	13.327(18)	18.830(63)	5.87(3)	22.0(1.3)	0.0040(11)	0.0121(7)
**4**	1.64(11)	13.273(17)	18.695(38)	5.82(9)	20.6(1.1)	0.0046(11)	0.0124(8)
**5**	1.59(11)	13.266(20)	18.687(83)	5.82(13)	19.4(1.1)	0.0048(13)	0.0126(9)
**6**	1.74(11)	13.262(18)	18.662(79)	5.82(14)	18.8(1.0)	0.0046(13)	0.0117(9)
**7**	1.42(9)	13.238(22)	18.489(95)	5.74(78)	18.4(1.1)	0.0045(15)	0.0077(9)
**8**	1.22(8)	13.232(27)	18.387(83)	5.8(1.0)	18.1(1.2)	0.0040(16)	0.0076(9)
**9**	1.18(8)	13.240(24)	17.834(73)	5.76(87)	19.5(1.4)	0.0043(17)	0.0090(8)
**10**	1.25(8)	13.235(24)	17.607(71)	5.6(1.1)	18.2(1.2)	0.0036(12)	0.0068(8)
**20**	1.64(10)	13.245(24)	17.524(72)	5.77(65)	18.3(1.1)	0.0059(13)	0.0141(9)
**30**	1.30(9)	13.200(27)	17.444(84)	5.7(1.1)	15.9(1.2)	0.0037(16)	0.0067(10)
**60**	1.11(7)	13.237(31)	17.641(60)	5.7(1.1)	23.3(1.8)	0.0051(12)	0.0052(10)

Time (min)	Sn x	Sn z	S1 z	S2 x	S2 z	S3 x	S3 z	Red Chi sq	Rw
**0**	0.4233(4)	0.4946(8)	0.573(4)	0.773(1)	0.454(3)	0.489(1)	0.590(2)	0.0706	0.4273
**1**	0.4232(5)	0.4950(10)	0.580(3)	0.774(1)	0.455(3)	0.491(1)	0.589(2)	0.0575	0.3999
**2**	0.4233(4)	0.4931(7)	0.579(2)	0.772(1)	0.447(2)	0.490(1)	0.589(2)	0.1143	0.4692
**3**	0.4231(5)	0.4950(11)	0.580(3)	0.774(2)	0.454(3)	0.491(1)	0.589(2)	0.0514	0.3882
**4**	0.4234(5)	0.4943(11)	0.578(2)	0.774(2)	0.454(3)	0.491(1)	0.585(2)	0.0604	0.3946
**5**	0.4234(5)	0.4944(13)	0.013(2)	0.774(2)	0.454(4)	0.491(1)	0.586(2)	0.0530	0.3866
**6**	0.4234(5)	0.4943(13)	0.579(2)	0.774(2)	0.454(3)	0.491(1)	0.585(2)	0.0594	0.3884
**7**	0.4234(6)	0.4938(15)	0.581(3)	0.774(2)	0.454(3)	0.491(1)	0.592(2)	0.0590	0.4134
**8**	0.4233(7)	0.4851(16)	0.576(3)	0.770(2)	0.435(3)	0.489(2)	0.596(2)	0.0627	0.4291
**9**	0.4229(7)	0.4893(17)	0.583(2)	0.772(2)	0.443(3)	0.490(2)	0.599(2)	0.0587	0.4228
**10**	0.4232(6)	0.4873(12)	0.583(3)	0.773(2)	0.443(3)	0.489(1)	0.601(1)	0.0594	0.4177
**20**	0.4236(6)	0.4854(13)	0.574(3)	0.772(3)	0.440(4)	0.488(2)	0.594(1)	0.0580	0.4225
**30**	0.4237(6)	0.4845(16)	0.581(3)	0.774(2)	0.441(4)	0.487(2)	0.602(1)	0.0681	0.4498
**60**	0.4233(5)	0.4883(12)	0.583(3)	0.773(1)	0.443(2)	0.489(1)	0.600(2)	0.0665	0.4476

Table S2: Refinement parameters of trenH-SnS-1 fits using isotropic atomic displacement parameters.

4.4 trenH-SnS-1, anisotropic atomic displacement model

Time (min)	Scale factor	a (Å)	c (Å)	Delta2	SP Dia (Å)	U_{11}(Sn) (Å^2)	U_{11}(S) (Å^2)	U_{33}(Sn) (Å^2)	U_{33}(S) (Å^2)
0	0.901(5)	13.285(12)	18.97(7)	5.72(38)	66.7(3.0)	0.0053(5)	0.0079(30)	0.057(21)	0.0153(18)
1	0.987(6)	13.259(14)	18.85(9)	5.67(40)	37.3(1.6)	0.0045(5)	0.0042(27)	0.086(36)	0.0033(66)
2	1.178(6)	13.347(10)	19.00(6)	5.73(11)	52.1(2.1)	0.0043(4)	0.0081(28)	0.244(70)	0.0019(39)
3	1.052(6)	13.304(16)	18.80(15)	5.71(27)	30.7(1.3)	0.0039(6)	0.0067(38)	0.134(72)	0.0058(12)
4	1.112(6)	13.243(15)	18.59(13)	5.70(19)	33.5(1.1)	0.0042(5)	0.0055(33)	0.218(91)	0.0056(10)
5	1.115(7)	13.231(18)	18.29(20)	5.74(18)	28.8(1.1)	0.0042(6)	0.0045(37)	0.24(11)	0.0111(16)
6	1.182(7)	13.245(15)	17.46(11)	5.82(12)	32.7(1.0)	0.0042(4)	0.0052(31)	0.46(16)	0.0023(57)
7	1.150(8)	13.212(20)	17.46(13)	5.66(26)	25.1(1.1)	0.0038(5)	0.0070(36)	0.178(94)	0.0023(62)
8	1.727(11)	13.253(26)	17.44(8)	5.83(13)	18.2(1.2)	0.0047(6)	0.0090(40)	0.144(10)	0.0079(17)
9	1.146(7)	13.206(22)	17.47(16)	5.76(51)	22.6(1.4)	0.0036(6)	0.0076(38)	0.099(61)	0.0008(88)
10	1.199(8)	13.227(23)	17.52(18)	5.79(52)	20.8(1.3)	0.0034(6)	0.0065(35)	0.102(65)	0.0011(84)
20	1.205(8)	13.197(30)	17.56(25)	5.79(48)	18.4(1.1)	0.0035(6)	0.0043(35)	0.111(93)	0.0062(17)
30	1.266(9)	13.190(30)	17.54(23)	5.67(55)	17.1(1.1)	0.0033(6)	0.0034(33)	0.105(88)	0.0031(13)
60	1.438(10)	13.199(24)	17.67(22)	5.81(14)	22.3(1.7)	0.0060(6)	0.0078(43)	0.063(45)	0.0664(73)

Time	Sn x	Sn z	S1 z	S2 x	S2 z	S3 x	S3 z	Red Chi sq	Rw
0	0.4234(5)	0.4940(3)	0.575(4)	0.770(2)	0.441(3)	0.489(2)	0.593(2)	0.0685	0.4209
1	0.4235(5)	0.4952(3)	0.577(4)	0.768(1)	0.439(2)	0.490(1)	0.593(2)	0.0593	0.4060
2	0.4234(4)	0.4956(3)	0.578(4)	0.766(1)	0.435(2)	0.489(1)	0.594(2)	0.1035	0.4463
3	0.4230(6)	0.4891(4)	0.576(5)	0.769(2)	0.437(3)	0.490(2)	0.594(2)	0.0567	0.4075
4	0.4233(5)	0.4889(3)	0.577(5)	0.769(2)	0.435(3)	0.490(2)	0.595(2)	0.0689	0.4213
5	0.4232(6)	0.4865(4)	0.571(8)	0.770(2)	0.433(4)	0.489(2)	0.597(3)	0.0594	0.4094
6	0.4232(4)	0.4898(4)	0.580(5)	0.774(2)	0.439(3)	0.490(2)	0.601(2)	0.0660	0.4093
7	0.4233(5)	0.4863(5)	0.578(5)	0.774(2)	0.440(4)	0.488(2)	0.601(2)	0.0571	0.4064
8	0.4232(6)	0.4864(4)	0.574(5)	0.773(3)	0.441(6)	0.487(2)	0.597(3)	0.0475	0.3733
9	0.4232(6)	0.4232(5)	0.578(5)	0.775(2)	0.443(4)	0.488(2)	0.602(2)	0.0540	0.4053
10	0.4232(6)	0.4857(5)	0.579(5)	0.774(2)	0.442(4)	0.489(2)	0.602(2)	0.0566	0.4074
20	0.4239(6)	0.4839(5)	0.577(7)	0.773(3)	0.437(6)	0.487(3)	0.602(2)	0.0653	0.4479
30	0.4237(6)	0.4836(5)	0.577(7)	0.773(3)	0.436(5)	0.487(2)	0.602(2)	0.0673	0.4461
60	0.4240(6)	0.4834(3)	0.573(4)	0.773(3)	0.442(8)	0.486(2)	0.595(2)	0.0591	0.4218

Table S3: Refinement parameters of trenH-SnS-1 fits using anisotropic atomic displacement parameters.

Time	c (Å), PXRD	U_{33}(Sn) (Å^2), free	U_{33}(Sn) (Å^2), fixed
0	18.97	0.057(21)	0.057(19)
1	17.08	0.086(36)	0.99(39)
3	16.30	0.134(72)	0.54(28)
5	16.06	0.24(11)	1.06(70)
10	15.75	0.102(65)	0.37(26)
20	15.61	0.111(93)	0.16(32)
60	15.63	0.063(45)	0.27(17)

Table S4: Anisotropic displacement parameters for Sn (U_{33}) from refinements with freely refined c-axes, or c-axes fixed at values obtained from PXRD.

5. Total scattering data and PDFs of trenH-SnS-1, stirring vs. no stirring

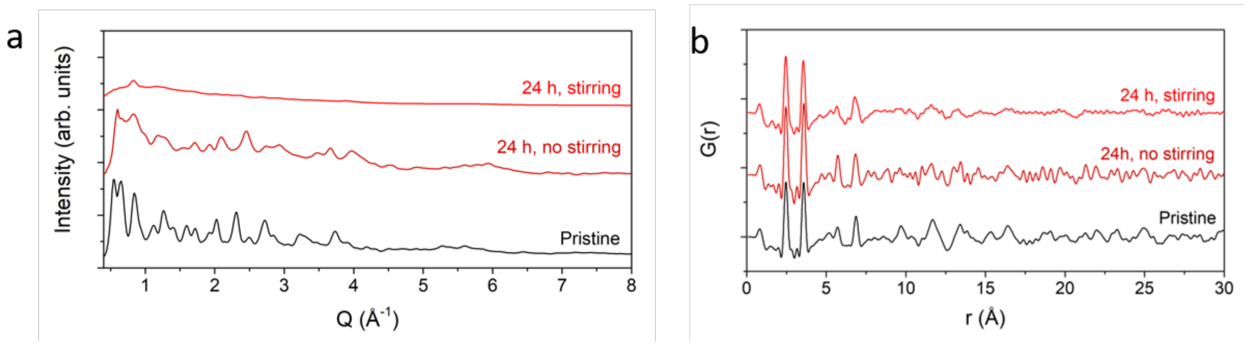

Figure S10: a) Synchrotron X-ray total scattering patterns and b) PDFs of trenH-SnS-1 after 24 h of dispersion in H_2O with and without magnetic stirring.

Total scattering patterns of pristine, non-stirred and stirred samples (in water 24 h) of trenH-SnS-1 (Fig. S10a). As the non-stirred sample contains a larger amount of precursor SnO_2 (different trenH-SnS-1 batches) and thus some peaks related to this phase, we will only compare $I(Q)$ at $Q < 1.8$ Å^{-1}, where no SnO_2 peaks are present (Baur & Khan, 1971). Evidently, more peaks remain in the non-stirred sample compared to that of the stirred sample, indicating a higher degree of order remaining in the non-stirred sample. The PDFs of the two samples (Fig. S10b) show retention of the local $[Sn_3S_7^{2-}]_n$ structure. In the PDF of the non-stirred sample, peaks related to the SnO_2 impurity is mainly observable in the high-r region (r > 10 Å), where the thiostannate correlations are weak in the water-treated sample.

6. Correlation sorting script and additional modelling

6.1 Correlation sorting script

A correlation-sorting MATLAB script was written as a tool to visualize refined correlations against the experimental PDFs. Output from refinements in PDFgui is used as input for the visualization script.

When using a cif-file in PDFgui as a structural starting model for PDF data analysis, an expansion of the structure from the original space group (here $P6_3/mmc$) to P1 is made, such that each atom in the unit cell is given a unique index. There are two thiostannate layers in one unit cell of AEPz-SnS-1 and trenH-SnS-1, and the unique atomic ID (*e.g.* "SN (#1)") allows identifying in which layer an atom in question is placed. This allows distinguishing between bonds/correlations within the layers ("intralayer correlations") and between different layers ("interlayer correlations").

The bond correlation output from PDFGui has the format:
SN (#1) - S (#18) = 2.40104 (0.0975516) Å

From the PDFgui expansion it is known that the atoms "SN (#1)" and "S (#18)" are in the same layer, and that the distance between the atoms is 2.40104 Å, with an error of 0.098 Å. By a "string compare", the script recognizes a correlation between Sn(1) and S(18), and that both atoms are within the same layer.
In addition to identifying (1) the correlation length and (2) whether the bond is intralayer or interlayer, we (3) apply a weighting to the correlation reflecting the scattering power of the two atoms in the correlation pair. Thereby, a Sn-Sn correlation will be weighted higher than an S-S correlation in the histogram. In the above example, a weighting is added by: $Z(\text{Sn}) * Z(\text{S}) = 50 * 16 = 800$. An S-S correlation will use the weighting of 16^2, where Sn-Sn uses 50^2. Each weighted correlation length is saved in a list for plotting in a histogram.

A bar in the resulting histograms is a sum of the weighted multiplicity of different correlations within the distance interval defined as one bar. The script identifies and sorts all correlations between 0-14.5 Å, while it (in its current form) is unable to extend beyond 14.5 Å. At larger distances, there is a risk of mixing intra- and interlayer correlations, as the atomic ID system will start to repeat itself, as layer *n* and *n*+2 are equivalent in the structure (as there are two thiostannate layers in one unit cell).

6.2 Debye refinements and simulations

In addition to the data analysis methods presented in the manuscript we performed Debye refinements of the PDF data in the program Diffpy-CMI by using the structure file obtained from the refinements in PDFgui. By doing so, we aimed at obtaining information on the crystallite shape and size. Only one Atomic Displacement Parameter (ADP) of S and Sn was varied (*i.e.* all Sn and all S atoms, respectively, were treated identically). The crystallite size was altered by introducing a supercell, and the shape and size were changed by varying the length along the *a*, *b* and *c* unit cell axes. The best refinement was evaluated from the lowest R_w factor. This method suggested crystallite sizes of 1-4 unit cells. As this result is unphysical (*e.g.* PXRD of the pristine samples reveal microsized crystallites), the method was not explored further.

In a different approach to describe the data, PDFs were calculated in Diffpy-CMI by varying the crystallite sizes and comparing the resulting PDFs to the experimental data. We calculated a series of PDFs where only the layer size (dimension in the *ab*-plane) was changed (while the size along *c* was fixed). This series was complemented by a series of simulated PDFs in which only the number of layers along *c* was changed. By comparing the two series of patterns, we aimed at identifying peaks that only changed in either case. The calculated PDFs were compared to the experimental data. However, both data series presented changes in the PDF intensities at similar distances, which complicated the peak assignment for both samples.

7. NMR spectroscopy

7.1 Solid state NMR

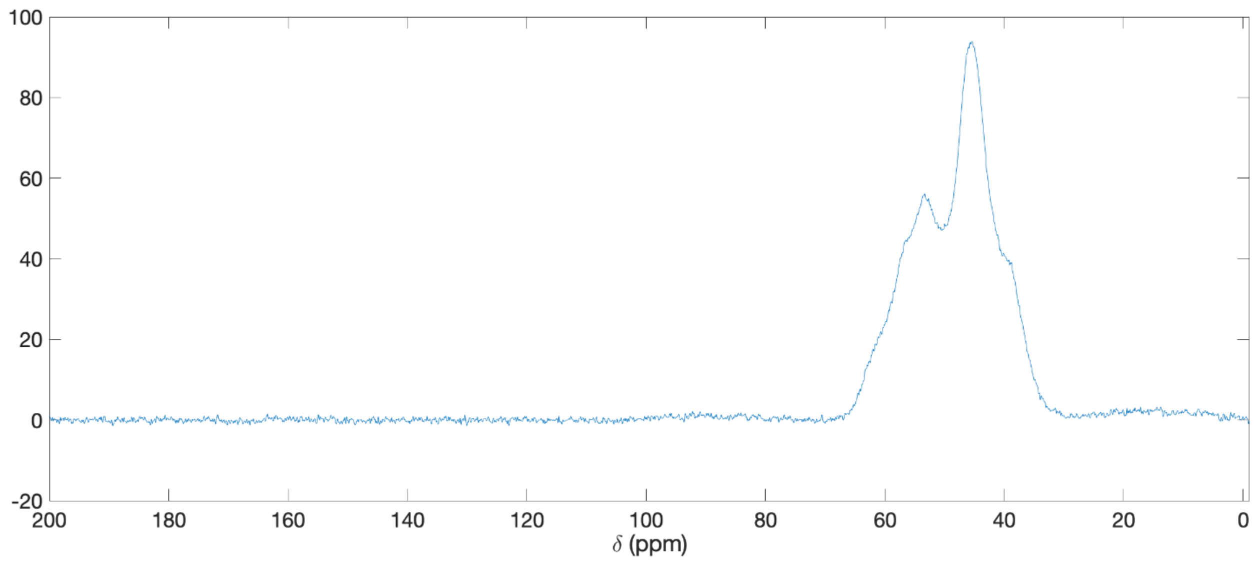

Figure S11: Solid-state $^{13}C\{^1H\}$ CP/MAS NMR spectrum of pristine AEPz-SnS-1

7.2 Liquid state NMR

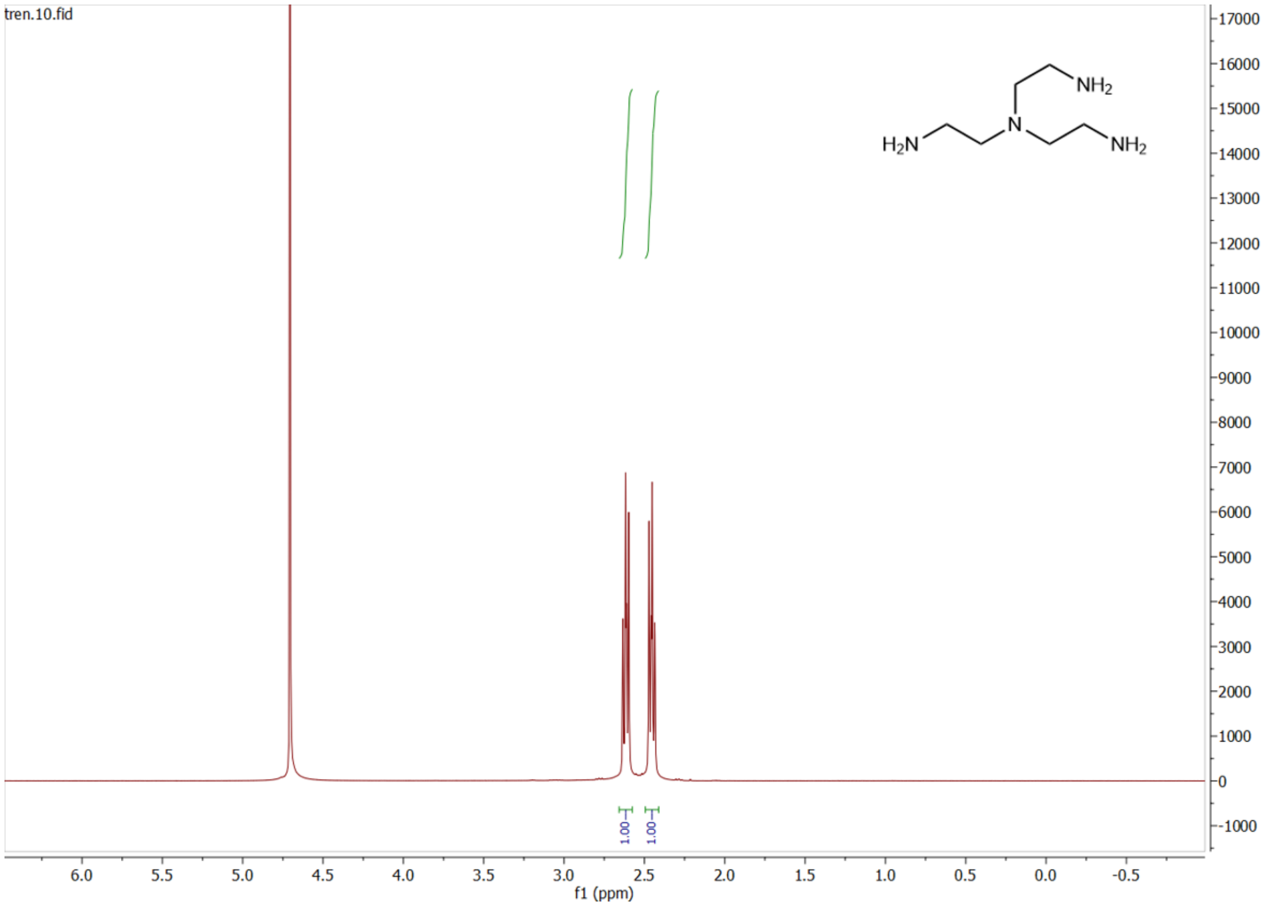

Figure S12: Liquid-phase ^1H NMR spectrum of tren (400 MHz, Avance III NMR spectrometer, D_2O).

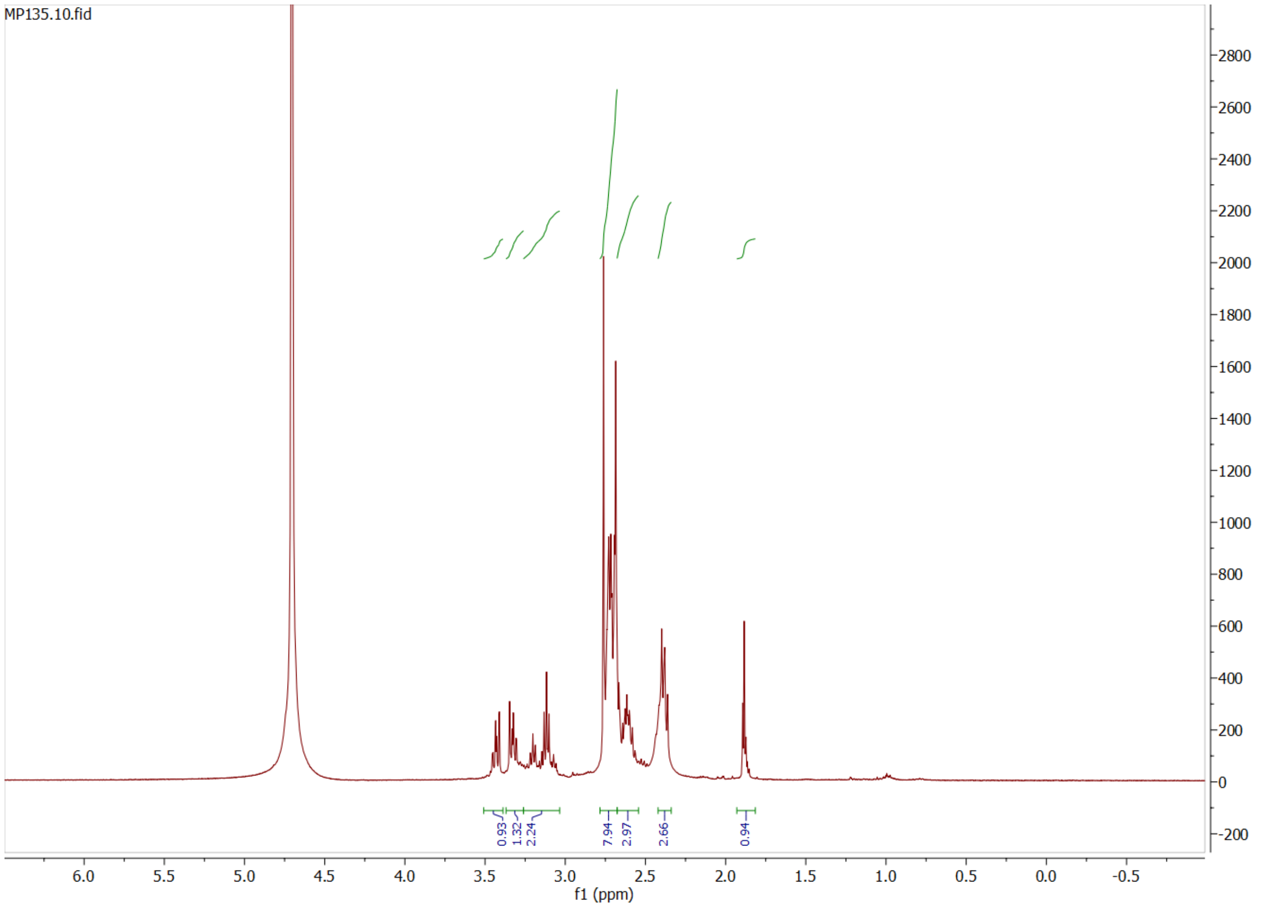

Figure S13: Liquid-phase ^{1}H NMR of leached amine species obtained by dispersion of trenH-SnS-1 in D_2O for 24 h (400 MHz, Avance III NMR spectrometer, D_2O).

8. TEM

TEM images were acquired on a Tecnai Spirit electron microscope equipped with a TWIN lens system operating at 120 kV, and using a Veleta CCD side mounted camera. TEM reveals formation of small particles in addition to larger particles identified by SEM (manuscript Fig. 4).

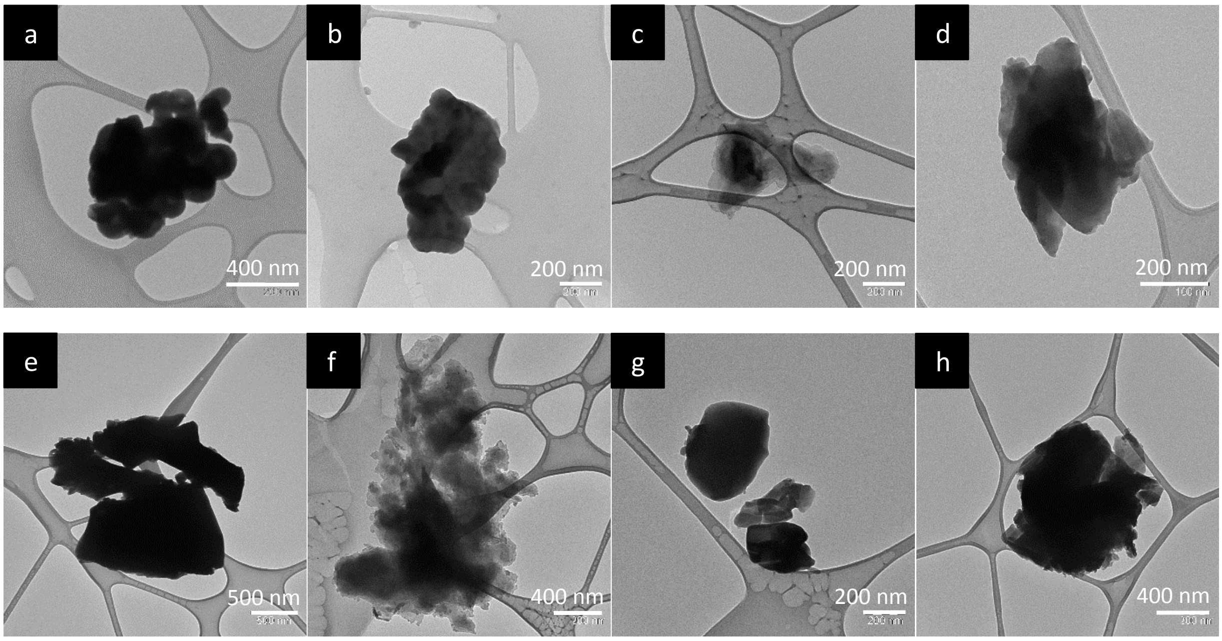

Figure S14: TEM images of water-treated R-SnS-1 compounds. a-d) AEPz-SnS-1 (24 h), e-h) trenH-SnS-1 (1 h).

9. XPS – spectra and atomic concentrations

Representative XPS spectra are shown in Fig. S15-S18. The relative atomic concentrations were determined using a Shirley background and deconvolution.

In the S 2p spectra of both pristine samples (Fig. S15c and S17c, respectively) an additional peak is observed besides the sulfide peak from the $[Sn_3S_7^{2-}]_n$ layers. The additional peak corresponds to oxidized sulfur (possibly sulfate) (Moulder & Chastain, 1992) and disappears by water treatment. The two Sn 3d peaks arise from the spin-orbit coupling (3d 5/2 and 3d 3/2) and are assigned to Sn^{4+}, as confirmed by literature (Price *et al.*, 1999, Hyeongsu *et al.*, 2018, He *et al.*, 2013).

All N is spectra present multiple components, indicative of multiple nitrogen sites. In Fig. S15e and S16e, the spectra of pristine and a water treated sample of trenH-SnS-1 are seen. Two components have been fitted, at binding energies of 399.8 and 401.7 eV (area ratio of 2.3:1 for the pristine sample) assigned to primary and tertiary amines, respectively, of the tren molecule. In the post water treatment, the primary-to-tertiary amine ratio has decreased to 1.3:1. Conversely, the N peaks (tertiary and primary/secondary amine) from AEPz, in Fig. S17 and S18, almost retains the same area ratio of 1:1.8 for the pristine sample and 1:1.7 for the water treated sample.

In all C spectra three components are found at binding energies of 285.5, 286.4 and 288.3 eV, which we suggest to arise from the C sp^3, C-N/C-O and carbonyl groups, due to the tren/AEPz molecules and surface contaminants.

The O 1s spectra have not been deconvoluted owing to the high contamination of the sample surface.

In Table S5-8 the areas of the integrated peaks are tabulated along with their respective binding energies. Table S9 displays the averaged composition of the samples. The composition presented in the manuscript is based on the values in Table S9.

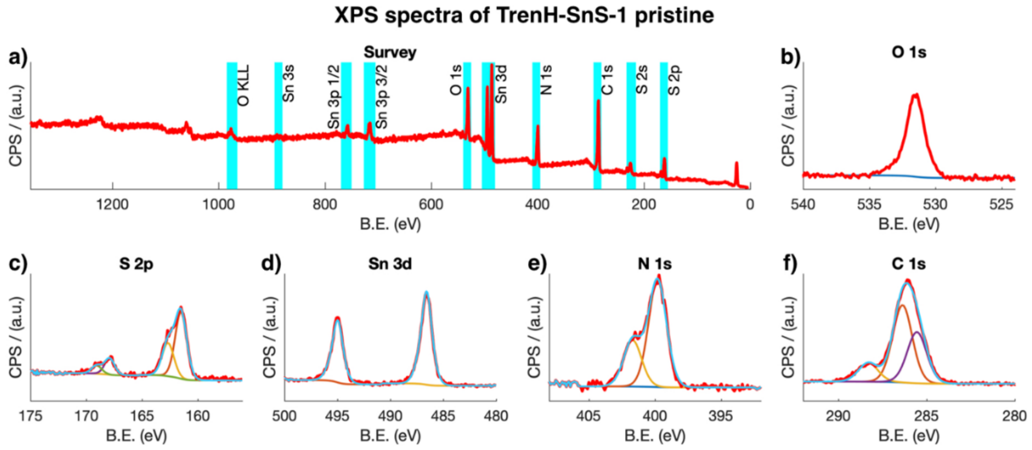

Figure S15: XPS spectra of the pristine trenH-SnS-1. a) Survey spectrum with peak identification of S, C, N, Sn and O. b-f) high resolution spectra of O, S, Sn, N and C, respectively. All have been fitted with a Shirley background.

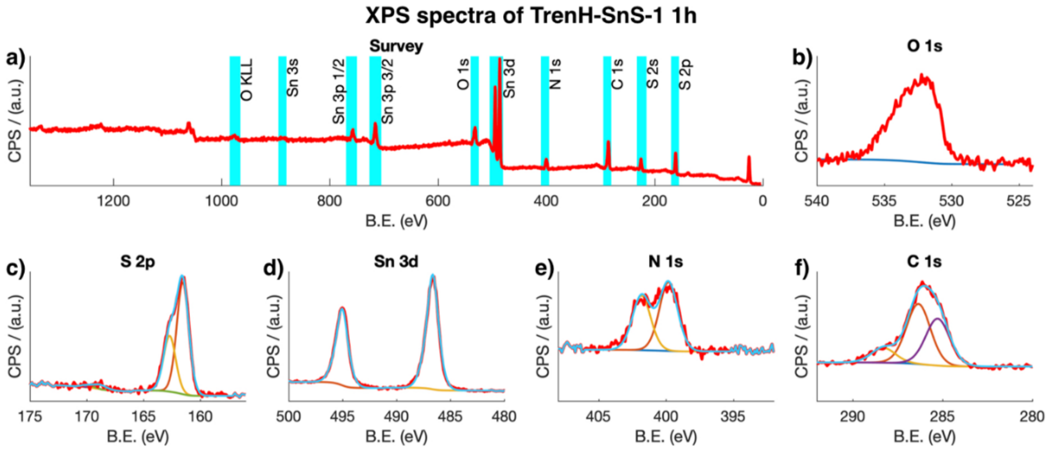

Figure S16: XPS spectra of the 1 h trenH-SnS-1. A) Survey spectrum with peak identification of S, C, N, Sn and O. b-f) high resolution spectra of O, S, Sn, N and C respectively. All have been fitted with a Shirley background.

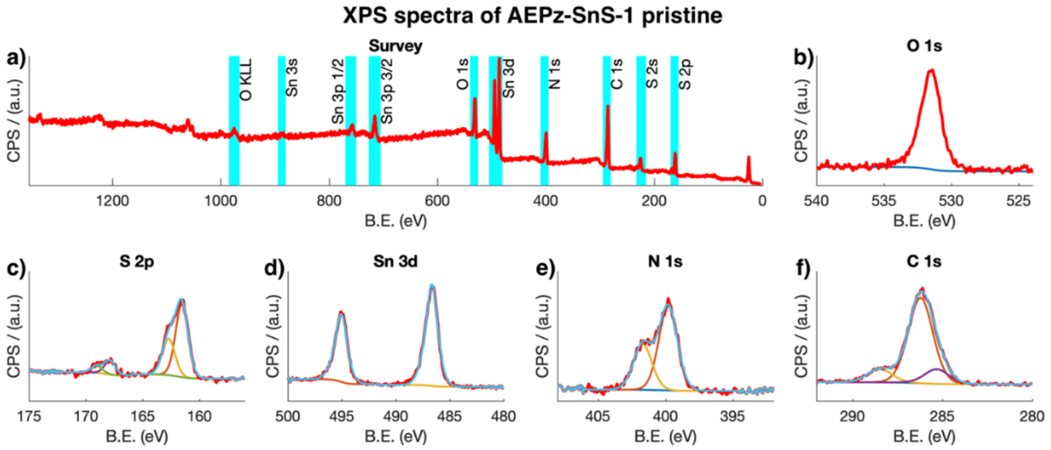

Figure S17: XPS spectra of the pristine AEPz-SnS-1. A) Survey spectrum with peak identification of S, C, N, Sn and O. b-f) high resolution spectra of O, S, Sn, N and C respectively. All have been fitted with a Shirley background.

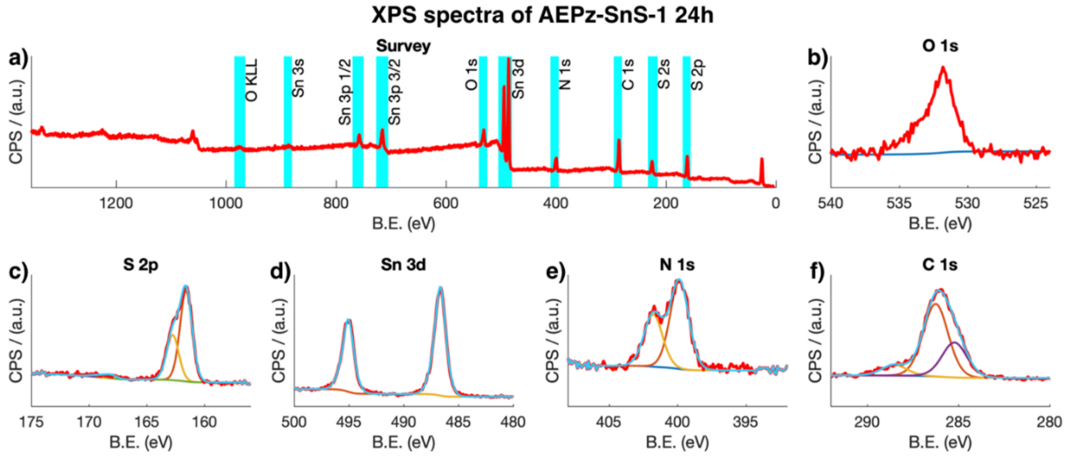

Figure S18: XPS spectra of the 24h AEPz-SnS-1. a) Survey spectrum with peak identification of S, C, N, Sn and O. b-f) high resolution spectra of O, S, Sn, N and C respectively. All have been fitted with a Shirley background.

	Spot 1		Spot 2		Spot 3	
	**BE (eV)**	**At%**	**BE (eV)**	**At%**	**BE (eV)**	**At%**
C (1s)	286.10	53.09	285.99	52.73	286.20	53.35
O (1s)	531.40	14.51	531.59	16.23	531.70	15.38
N (1s)	399.70	21.40	400.09	20.83	400.00	20.31
Sn (3d)	486.60		486.59		486.60	
Sn (3d)	495.00	4.15	494.99	3.90	494.90	4.31
S (2p) SnS	161.50	5.66	161.49	5.13	161.70	5.50
S (2p) SO_x	167.80	1.18	167.59	1.18	168.00	1.15

Table S5: Atomic concentrations and binding energies of individually measured spots for trenH-SnS-1, pristine.

	Spot 1		Spot 2		Spot 3	
	**BE (eV)**	**At%**	**BE (eV)**	**At%**	**BE (eV)**	**At%**
C (1s)	286.13	48.50	286.10	47.77	285.70	54.90
O (1s)	532.23	16.24	532.20	16.68	531.99	15.00
N (1s)	399.63	12.77	400.00	12.62	399.69	10.79
Sn (3d)	486.53		486.60		486.69	
Sn (3d)	495.03	10.15	495.10	10.07	494.99	8.31
S (2p) SnS	161.43	11.86	161.50	12.39	161.49	10.25
S (2p) SO_x	168.73	0.50	169.20	0.47	168.79	0.75

Table S6: Atomic concentrations and binding energies of individually measured spots for trenH-SnS-1, 1h.

	Spot 1		Spot 2		Spot 3	
	**BE (eV)**	**At%**	**BE (eV)**	**At%**	**BE (eV)**	**At%**
C (1s)	286.10	53.13	286.18	52.98	286.21	53.49
O (1s)	531.40	14.36	531.48	14.02	531.31	16.98
N (1s)	399.80	19.60	399.88	19.30	399.71	16.92
Sn (3d)	486.70		486.68		486.51	
Sn (3d)	495.10	5.13	494.98	5.21	494.91	4.86
S (2p) SnS	161.50	6.71	161.58	6.97	161.61	6.59
S (2p) SO_x	167.80	1.06	167.98	1.52	167.71	1.15

Table S7: Atomic concentrations and binding energies of individually measured spots for AEPz-SnS-1, pristine.

	Spot 1		Spot 2		Spot 3	
	**BE (eV)**	**At%**	**BE (eV)**	**At%**	**BE (eV)**	**At%**
C (1s)	285.90	51.19	285.80	51.72	285.86	50.23
O (1s)	531.80	11.23	532.10	11.05	531.86	11.73
N (1s)	400.00	15.03	399.70	13.87	399.86	14.18
Sn (3d)	486.60		486.50		486.55	
Sn (3d)	494.90	9.39	494.90	10.19	495.05	10.17
S (2p) SnS	161.60	12.41	161.50	12.85	161.46	12.94
S (2p) SO_x	169.40	0.74	168.30	0.31	168.56	0.74

Table S8: Atomic concentrations and binding energies of individually measured spots for AEPz-SnS-1, 24 hrs.

	AEPz-SnS-1 Pristine At%	AEPz-SnS-1 24 h At%	trenH-SnS-1 pristine At%	trenH-SnS-1 1 h At%
**C**	53.20 ± 0.15	51.05 ± 0.44	53.06 ± 0.18	50.39 ± 2.26
**O**	15.12 ± 0.94	11.34 ± 0.20	15.37 ± 0.50	15.97 ± 0.50
**N**	18.61 ± 0.85	14.36 ± 0.35	20.85 ± 0.31	12.06 ± 0.64
**Sn**	5.07 ± 0.11	9.92 ± 0.26	4.12 ± 0.12	9.51 ± 0.60
**S (SnS)**	6.76 ± 0.11	12.73 ± 0.16	5.43 ± 0.16	11.50 ± 0.64
**S (SO_x)**	1.24 ± 0.14	0.60 ± 0.14	1.17 ± 0.01	0.57 ± 0.09

Table S9: Results of all atom percentages of the XPS data determined from high resolution spectra.

10. Elemental analysis

CHNS (wt%)	AEPz-SnS-1 Theoretical	AEPz-SnS-1 Pristine	AEPz-SnS-1 24 h	TrenH-SnS-1 Theoretical	trenH-SnS-1 pristine	trenH-SnS-1 1 h
**C**	17.14	19.14 ± 0.05	13.18 ± 0.08	16.47	16.74 ± 0.10	12.38 ± 0.20
**H**	3.84	4.06 ± 0.22	3.19 ± 0.19	4.38	3.99 ± 0.22	2.99 ± 0.15
**N**	9.99	10.79 ± 0.01	8.03 ± 0.04	12.81	10.68 ± 0.07	7.76 ± 0.14
**S**	26.69	23.72 ± 0.50	26.87 ± 0.26	25.65	24.35 ± 0.32	27.68 ± 0.47

Table S10: Elemental analysis of AEPz-SnS-1 and trenH-SnS-1 pristine and water-treated for 1 hour. Theoretical values are based on a pristine sample with no contaminants and with two organic amines per ($Sn_3S_7^{2-}$)$_n$.

11. Kubelka-Munk functions of diffuse reflectance spectroscopy

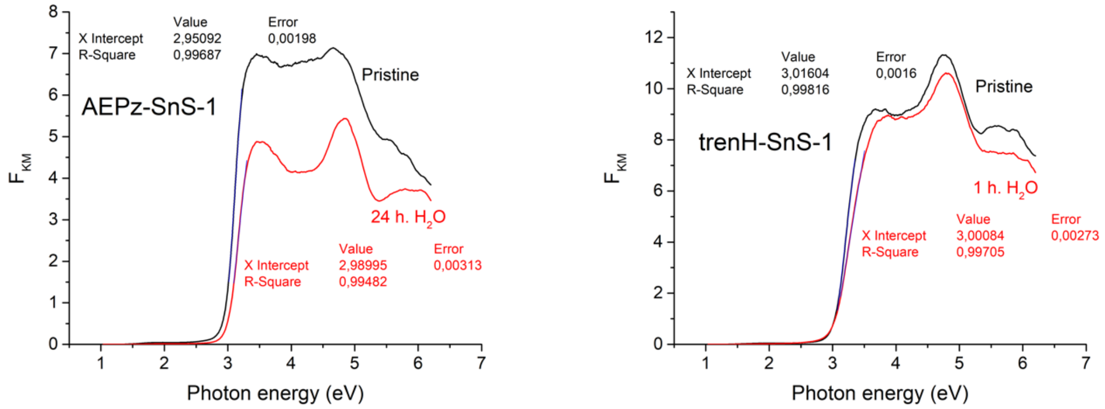

Figure S19: Reflectance data converted to the Kubelka-Munk function, $F_{KM} = \frac{(1-R)^2}{2R}$, where R is the reflectance. Band gaps are determined as the interception between the linear extrapolation of the absorption edge and the x-axis.

12. References

Baur, W. H. & Khan, A. A. (1971). *Acta Crystallographica Section B* **27**, 2133-2139.

Filsø, M. Ø., Chaaban, I., Al Shehabi, A., Skibsted, J. & Lock, N. (2017). *Acta Crystallogr., Sect. B* **73**, 931-940.

He, M., Yuan, L.-X. & Huang, Y.-H. (2013). *RSC Adv.* **3**, 3374-3383.

Hyeongsu, C., Jeongsu, L., Seokyoon, S., Juhyun, L., Seungjin, L., Hyunwoo, P., Sejin, K., Namgue, L., Minwook, B., Seung-Beck, L. & Hyeongtag, J. (2018). *Nanotechnology* **29**, 215201.

Moulder, J. F. & Chastain, J. (1992). *Handbook of x-ray photoelectron spectroscopy: A reference book of standard spectra for identification and interpretation of xps data*. Physical Electronics Division, Perkin-Elmer Corporation.

Price, L. S., Parkin, I. P., Hardy, A. M. E., Clark, R. J. H., Hibbert, T. G. & Molloy, K. C. (1999). *Chem. Mater.* **11**, 1792-1799.
